# High-Throughput Screening of Five Compound Libraries for Anthelmintic Activity and Toxicity Leads to the Discovery of Two Flavonoid Compounds

**DOI:** 10.3390/ijms26041595

**Published:** 2025-02-13

**Authors:** Giulio Galli, Marta Ruiz-Somacarrera, Laura González del Palacio, Estela Melcón-Fernández, Rubén González-Pérez, Carlos García-Estrada, Maria Martinez-Valladares, Rafael Balaña-Fouce

**Affiliations:** 1 Departamento de Ciencias Biomédicas, Facultad de Veterinaria, Universidad de León, Campus de Vegazana s/n, 24071 León, Spain; ggal@unileon.es (G.G.); emelf@unileon.es (E.M.-F.); rgonp@unileon.es (R.G.-P.); cgare@unileon.es (C.G.-E.); 2 Departamento Sanidad Animal, Instituto de Ganadería de Montaña, CSIC-Universidad de León, Grulleros, 24346 León, Spain; mruis@unileon.es (M.R.-S.); lgonzp@unileon.es (L.G.d.P.); 3 Instituto de Biomedicina (IBIOMED), Universidad de León, Campus de Vegazana s/n, 24071 León, Spain

**Keywords:** anthelmintic, chalcone derivatives, tolfenpyrad, octenidine, toxicity, selective index, organoid, high-throughput screening

## Abstract

Gastrointestinal nematode infections (GINs) in ruminants are a major constraint to efficient livestock production worldwide. Currently, only a limited number of anthelmintic drugs are available for the control of these infections, but their widespread use in preventive deworming campaigns and the incorrect administration of the drugs are responsible for the emergence of resistance. Therefore, new anthelmintic drugs are urgently needed. However, drug discovery methods for new anthelmintics based on GINs isolated from ruminants often have low throughput. In this study, a screening of five commercial collections of chemical compounds, including one collection of anti-infective drugs, three plant-based natural product collections, and one collection from the FDA-approved Chinese *Pharmacopoeia*, with a total of 2228 molecules, have been carried out in a high-throughput format. In the single slot screen, 32 compounds (1.44% success rate) achieved a >70% motility inhibition rate. Of these, 10 are known anthelmintic drugs, while the remaining 22 were tested against *Haemonchus contortus* and a resistant strain of *Teladorsagia circumcincta*. Four compounds (two flavonoids, chalcone and trans-chalcone), and two anti-infectives (octenidine and tolfenpyrad), showed anthelmintic activity with EC_50_ values below 20 µM, and were further tested for their safety against HepG2 spheroids and mouse intestinal organoids. Trans-chalcone and chalcone emerged as promising candidates for future development, showing selective indexes > 5, while tolfenpyrad and octenidine require careful evaluation due to their toxicity profiles.

## 1. Introduction

Helminthic parasitic diseases, caused by gastrointestinal nematodes (GINs), are among the most important health problems affecting livestock worldwide [1,2]. Parasites such as *Haemonchus contortus*, *Teladorsagia circumcincta*, *Trichostrongylus* spp. and others [3] infect the gastrointestinal tract of sheep, goats and other ruminants, causing serious health problems. The clinical manifestations of these infections include anemia, weight loss, reduced milk production and, in severe cases, death [4]. These diseases not only have an impact on animal welfare, but also cause significant economic losses in the agricultural sector, decreasing productivity and increasing the costs of animal health management [5]. Globally, helminth infections are ubiquitous, with varying prevalence depending on geographic and climatic conditions [6]. In regions with warm and humid climates, such as parts of the southern United States, Brazil, and sub-Saharan Africa, the prevalence of these parasites is particularly high, exacerbating their impact on livestock [2].

The use of anthelmintic drugs has been the main method to control parasitic nematode infections for a long time. However, the efficacy of these treatments is increasingly compromised by the growing problem of anthelmintic resistance. In recent decades, multiple resistance to several classes of anthelmintics, such as benzimidazoles, levamisole and macrocyclic lactones, was documented worldwide [7,8,9]. This increasing resistance threatens the sustainability of livestock production systems and, therefore, there is an urgent need to implement new control measures, and to identify and develop new compounds with anthelmintic activity to ensure the health and productivity of farm animals worldwide. Phenotypic screening with GINs for anthelmintics includes different techniques, such as egg hatching and motility assays of larval stages. However, these methods are difficult to adapt to high-throughput screening (HTS) techniques and often require infection from a live donor. In this context, *Caenorhabditis elegans* has emerged as a valuable surrogate organism for the screening of potential anthelmintic compounds. *C. elegans* is a free-living nematode that can be easily maintained and manipulated under laboratory conditions and with standard in vitro techniques, thus making it an accessible and effective tool for HTS platforms in anthelmintic drug discovery [10].

This nematode has a short generation time, low maintenance costs, long-term cryopreservation capability and allows for rapid propagation and experimentation [11,12]. In addition, *C. elegans* shares most of the conserved cellular processes with other metazoans, including parasitic nematodes, making it a relevant model for studying drug efficacy and mechanisms of action [13,14]. Several studies showed that compounds lethal to *C. elegans* are also lethal to parasitic nematodes, and more than half of the lethal compounds identified in a large-scale screen showed cross-species efficacy [14,15]. This suggests that *C. elegans* is a reliable surrogate for identifying compounds with broad-spectrum anthelmintic activity [4,10]. However, its use as a model organism in drug discovery against parasitic helminths also has some limitations. A major drawback is the relatively inefficient uptake of drugs due to the low permeability of the *C. elegans* cuticle to non-water-soluble compounds, as well as the selective uptake of drugs by the gut [13]. Furthermore, although *C. elegans* is genetically similar to parasitic nematodes, there are species-specific genes and unique parasitic pathways linked to virulence, which may lead to discarding potential anthelmintic compounds that are effective against parasites, but not on *C. elegans* itself [14,16].

A key step in the drug discovery process is to assess the safety of the lead compounds in host animals. To bridge this gap between initial screening and in vivo studies, advanced three-dimensional (3D) cell-culture systems such as liver spheroids and intestinal organoids emerged as promising alternative methods, since they more closely mimic the in vivo environment than traditional two-dimensional (2D) cell models [17,18]. Liver spheroids are aggregates of liver cells that maintain key liver functions such as drug metabolism and protein synthesis for prolonged periods [19,20,21], while intestinal organoids are miniaturized, self-organizing stem cell-derived tissues that reproduce the complex architecture and cellular diversity of the gut, thereby allowing for a more accurate modeling of intestinal absorption and metabolism [22,23,24,25,26]. These 3D systems offer significant advantages over 2D models, such as improved cell–cell and cell–matrix interactions, more physiologically relevant gene-expression profiles and the greater predictability of in vivo drug responses. As such, they are increasingly used in pharmacological/toxicological studies to assess the efficacy, safety and mechanisms of action of drugs in a more biologically relevant context [19,27,28].

Overall, this study aims to identify novel anthelmintic drug candidates by screening five commercial collections of compounds using *C. elegans* as a model organism through single-shot (SS) and dose–response (DR) assays. Effective compounds were tested in susceptible and resistant parasitic helminth strains, whereas toxicity was assessed in HepG2 spheroids and mouse intestinal organoids.

## 2. Results

### 2.1. HTS on C. elegans

**Figure 1:**
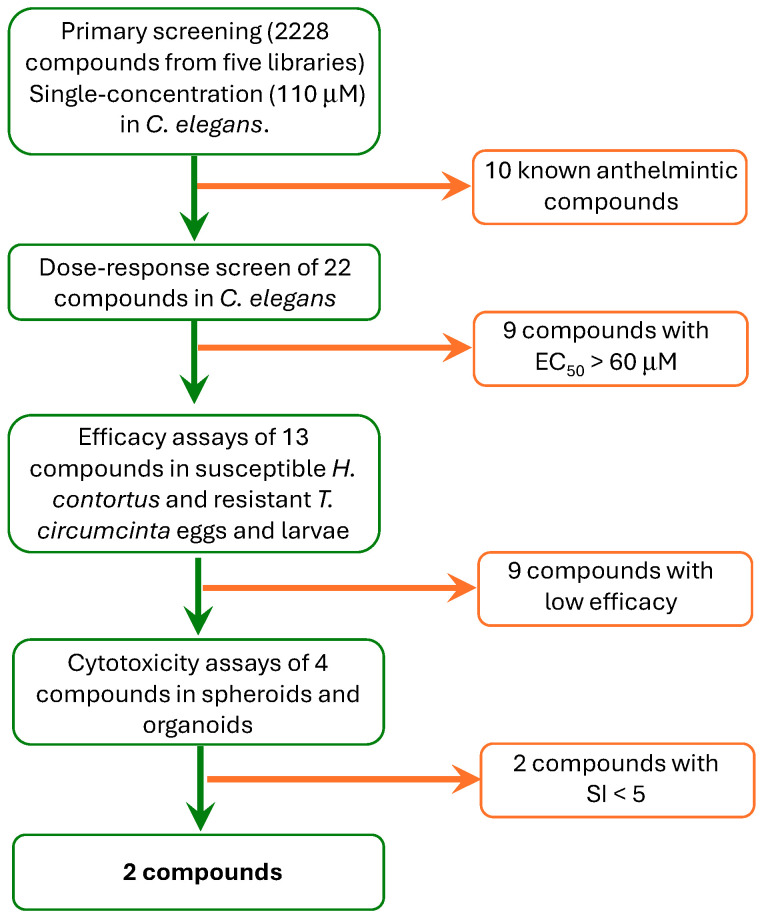
A cascade of events showing the screening strategy with the progression from in vitro assays in *C. elegans* to in vitro efficacy tests in parasitic worms and in vitro cytotoxicity tests in spheroids and organoids. A total of 2228 compounds included in the Anti-Infection Compound Library and four collections of plant-derived compounds (MedChemExpress) were evaluated.

The strategy followed to identify effective compounds against parasitic helminths is shown in Figure 1. For this purpose, five commercial drug collections from MedChemExpress (a total of 2228 compounds) were tested for their effects on the motility of *C. elegans* worms. Libraries (see Materials and Methods) included a repurposing collection of anti-infective drugs and four collections of plant-derived compounds, since plan- based agents have shown efficacy on other metazoa [29].

The SS assay at 110 μM in *C. elegans* was conducted to screen an initial set of 2228 molecules from five commercial compound libraries. To identify potential pre-hits, a motility inhibition value > 70% at 0 h or 24 h was established as the cut-off point. Inhibition at 0 h but not at 24 h was considered a sign of paralysis. On the other hand, inhibition at 24 h or at both time points was considered indicative of death. The quality of the HTS assay was assessed by measuring the signal-to-background ratio between negative and positive controls, establishing a Z′ value > 0.5 (Figure S1 and Table S1). Additionally, DR tests with known anthelmintics were performed to validate the assay (Figure S2). The calculated EC_50_ values for ivermectin, levamisol and moxidectin were 2.18 µM, 1.91 µM and 0.79 µM, respectively. The EC_50_ values found in the literature for these compounds were 0.19 µM [15], 6.4 µM [15] and 0.03 µM [16], respectively.

**Figure 2:**
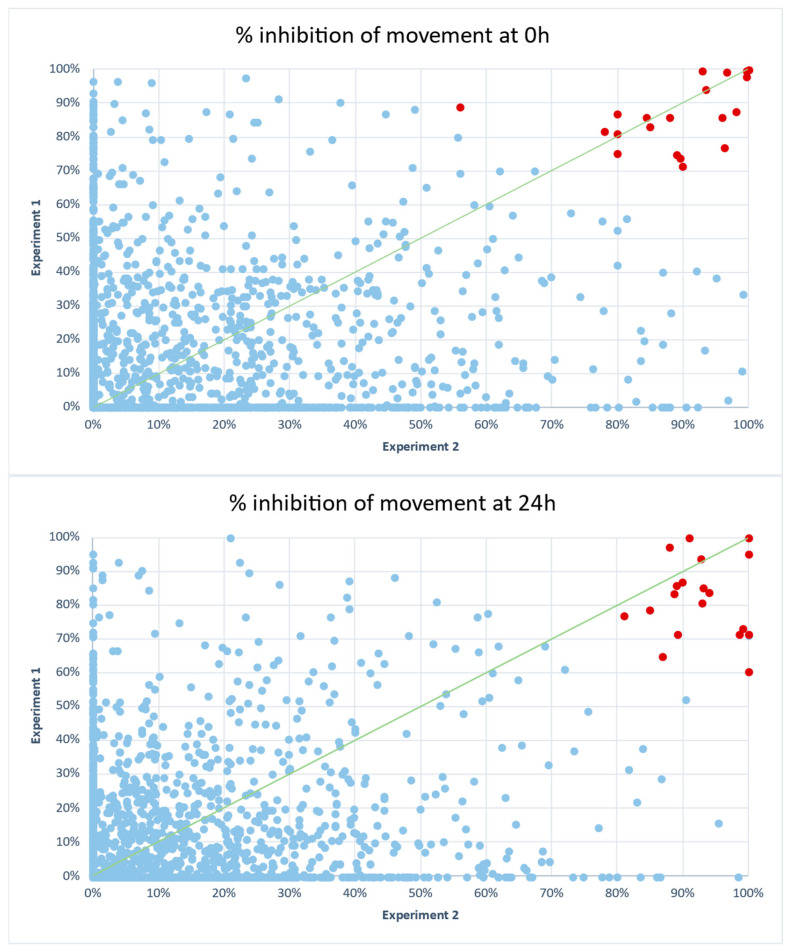
A screening of the 2228 compounds included in the Anti-Infection Compound Library (MedChemExpress) and natural products libraries on *C. elegans* motility after 0 h and 24 h in the presence of those compounds. Correlation plots were performed with data of two independent replicates of the *C. elegans* in vitro assay using each single-point compound at 110 μM. Each dot represents a single compound. Compounds with an average inhibition of at least 70% are shown in red, while inactive compounds are represented in blue. Green line represents ideal correlation.

The list of all 2228 compounds and relative inhibition rates are shown in Table S2. From these compounds, 32 (1.44%) pre-hits were identified meeting or exceeding the inhibition threshold (Figure 2 and Table 1).

In the Anti-Infection Compound Library, from the 23 compounds found to be active against *C. elegans* (hit rate: 4.7%), ten compounds with known anthelmintic activity, including macrocyclic lactones (avermectin B1, eprinomectin, moxidectin, milbemycin and ivermectin), imidothiazoles (levamisole and tetramisole), the salicylanilide oxyclozanide and pyrantel derivatives (pyrantel pamoate and pyrantel tartrate), were known anthelmintics drugs. These compounds were discarded from further analysis, although their identification demonstrated the robustness of the platform in detecting anthelmintic drugs. Considering only the active compounds not known to be in clinical use, the hit rate was 2.66% (13/489), the highest among all the libraries tested. Regarding the natural product libraries, the success rate was much lower. No hits were found in the alkaloids library (0/449), whereas three hits were found in the flavonoid (3/343), terpenoid (3/629) and the FDA-approved Traditional Chinese Medicine (3/318) libraries, respectively.

DR studies were carried out with these compounds (Figure S3), providing *p*-values < 0.05, R > 0.90 and the EC_50_ values and standard errors included in Table 1. Since nine compounds showed EC_50_ values above 60 µM, in order to minimize the number of false negatives, a threshold concentration of 60 µM was set, thus reducing the number of compounds that could be evaluated in the subsequent parasite assays from 23 to 13. In the Anti-Infection Library, only six of the selected compounds had an effect below the pre-established threshold of 60 µM. Thus, ethacridine lactate (CAS# 1837-57-6), ethacridine lactate monohydrate (CAS# 6402-23-9), octenidine (CAS# 70775-75-6), robenidine (CAS# 25875-51-8), salinomycin (CAS# 53003-10-4) and tolfenpyrad (CAS# 129558-76-5) were selected for further studies on parasitic nematodes susceptible and resistant to standard anthelmintics. Among the nine compounds identified in the four collections of natural products, two molecules (one terpenoid and one from the FDA-approved Traditional Chinese Medicine library) did not meet the criterion of having an EC_50_ < 60 µM, and were removed from further studies. Therefore, kuwanon G (CAS# 75629-19-5), chalcone (CAS# 614-47-1), trans-chalcone (CAS# 614-47-1), polygodial (CAS# 6754-20-7), 3,29-dibenzoyl rarounitriol (CAS# 873001-54-8), resveratrol (CAS# 501-36-0) and dioscin (CAS# 19057-60-4) were selected for further studies on parasitic helminths.

### 2.2. Study on Parasitic Helminths

The 13 active compounds identified in the *C. elegans* motility inhibition assay were tested against two species of parasitic helminths causing gastrointestinal problems in sheep. The compounds were tested at a single concentration of 100 μM against a susceptible strain of *H. contortus* and a resistant strain of *T. circumcincta*. The ovicidal (egg hatch inhibition; EHI) and larvicidal (larval migration inhibition test; LMIT) effects (Table 2) were determined by the corresponding assays, as detailed in Materials and Methods. Only the anti-infective agents tolfenpyrad and octenidine, as well as the flavonoids chalcone and trans-chalcone, gave rise to a >90% EHI or LMIT in susceptible *H. contortus* and resistant *T. circumcincta*. The effect of the other compounds was either negligible or too weak to be considered therapeutically relevant. Notably, octenidine was the only compound able to reduce the viability of both eggs and larvae in susceptible and resistant parasites by more than 90% across all tests. In contrast, the other compounds showed weaker effects, although all were effective as ovicide molecules in susceptible strains of *H. contortus* and in the resistant strain of *T. circumcincta* (Table 2).

The EC_50_ values for the EHI tests in both helminth species were determined using dose–response curves (Figure S3), showing a strong ovicidal effect of the four selected compounds in both susceptible and resistant strains. Unlike octenidine, the other three compounds had EC_50_ values below 10 μM, with tolfenpyrad displaying a remarkable ovicidal effect with an estimated EC_50_ of 0.2 μM (Table 3).

### 2.3. Cytotoxicity of the Hits in Liver Spheroids and Intestinal Organoids

To complete the study of the hits identified in the previous experiments, their in vitro safety was evaluated. Two different 3D models of acute in vitro toxicity tests were used: liver spheroids from HepG2 cells as a model for systemic toxicity, and mouse intestinal organoids from adult stem cells (enteroids) as a model for tolerance to the compounds after oral administration. Both models were validated by performing a Z′-factor assay, achieving values ≥ 0.5 (Figures S4 and S5 and Tables S3 and S4). The results in Table 3 show that the four hits exhibited minimal hepatotoxicity at the maximum concentration tested in HepG2 spheroids. Oral tolerance tests with mouse enteroids revealed the marked enterotoxicity of tolfenpyrad and high enterotoxicity of octenidine. In terms of selectivity, tolfenpyrad showed a high SI with HepG2 spheroids, but a very low SI with mouse organoids, thus highlighting its significant enterotoxicity. Octenidine showed a medium SI overall, but demonstrated a high SI when tested against *H. contortus* eggs. Chalcone showed high SI values, except in tests with the *T. circumcincta*-resistant strain. Trans-chalcone, on the contrary, showed consistently high or very high SI values in all tested models, underlining its potential as a selective and safe compound for future studies.

### 2.4. Predictive ADMET

**Figure 3:**
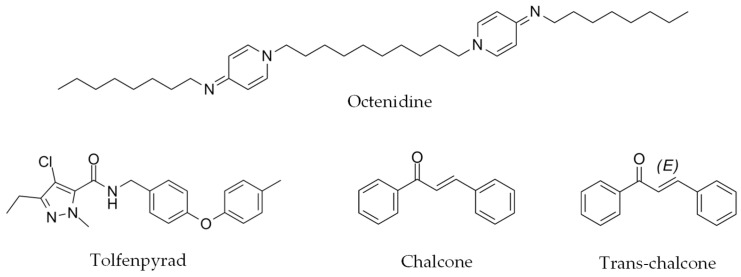
The chemical structure of octenidine, tolfenpyrad, chalcone and trans-chalcone.

Octenidine, tolfenpyrad, chalcone and trans-chalcone (Figure 3) were subjected to predictive pharmacokinetic studies.

Data, summarized in Table 4, suggest that tolfenpyrad does not violate any of Lipinski’s rules and may have good oral bioavailability. However, toxicity predictions by admetSAR 2.0 revealed a toxicological profile of concern, including hepatotoxicity, nephrotoxicity, respiratory and reproductive toxicity. Octenidine violates two of Lipinski’s rules due to its high molecular weight and high lipophilicity. The model predicts poor oral absorption, no blood–brain barrier penetration, and no interaction with key cytochrome P450 enzymes. ADMETsar 2.0 analysis suggested that octenidine may bind to the estrogen receptor and poses potential hepatotoxicity, mitochondrial toxicity and reproductive toxicity. In contrast, according to swissADME website, chalcone and trans-chalcone do not violate any Lipinski rules, showing moderate lipophilicity and low molecular weight, which suggest a fast absorption in the gastrointestinal tract. These two molecules have similarly favorable safety predictions, with no hepatotoxicity or nephrotoxicity, and with a negative result in the AMES test, indicating low mutagenic potential.

## 3. Discussion

The GINs of farm ruminants cause prevalent infections responsible for high economic losses worldwide. Phenotypic screening remains the most efficient method for identifying effective anthelmintics in drug discovery campaigns, since the knowledge of potential pharmaceutical targets in the parasites is still scarce. However, the challenge of requiring farm animals to complete the life cycle of parasitic helminths has led to the use of free-living surrogate species, such as *C. elegans*, which has emerged as an alternative model for initial HTS of compound libraries. The ease of reproducing its complete life cycle, the ability to establish HTS platforms in 96- or 384-well plate formats, the availability of powerful molecular, genetic and genomic tools, and the extensive online resources for studying physiological effects, gene functions and drug target identification, combined with automated larval movement detection, make *C. elegans* an invaluable model for the primary screening of anthelmintic drugs [10].

Salinas and Risi [14] observed that molecules lethal to *C. elegans* were up to fifteen times more likely to also kill GINs compared to randomly selected molecules. Moreover, nearly 40% of the compounds lethal to *C. elegans* were also lethal to the parasitic nematodes *Cooperia oncophora* and *H. contortus*. These findings confirm that *C. elegans* is a useful model for anthelmintic drug discovery, despite some limitations [13,30], including a significant false-negative hit rate [31] and the absence of virulence genes, which may hinder the identification of active molecules against parasitic nematodes. Shanley and colleagues [32] found that the hit rate in *C. elegans* was half of that observed in *H. contortus* (8 vs. 16 hits), highlighting some limitations of the *C. elegans* model. However, strong evidence suggests that many compounds with larvicidal and ovicidal activity against *C. elegans* also exhibit anthelmintic effects on target nematodes. Therefore, despite its drawbacks, the advantages of using *C. elegans* as a primary screening tool, such as its high-throughput capacity, low cost, and feasibility, far outweigh its disadvantages.

These advantages have led to the development of many HTS approaches using motility as the endpoint [4,15,16,31,33], while others rely on the microscopic observation of larvae after exposure to the compounds [34]. The hit search success rate is generally low (0.18–3.33%), similar to the 1.44% found in the current study. However, Risi and colleagues [15] reported a 16% success rate using a proprietary library specifically designed for anthelmintic research and an inhibition cutoff of 35%. When only compounds with >75% inhibition were considered, their success rate dropped to 3.43%, which is consistent with other studies. Toxicological evaluations in previous studies have included zebrafish assays and computational PROTOX software (v3.0) [15], as well as HepG2 cytotoxicity testing [32], and some tests are still in progress [16]. Unlike these studies, our work uniquely employed 3D cell cultures to assess the toxicity and tolerance of the identified compounds.

Among the compounds identified with anthelmintic activity, tolfenpyrad exhibited remarkable efficacy, which is consistent with other studies [15,32]. Risi and colleagues [15] calculated an EC_50_ of 3.6 µM in *C. elegans*, which is similar to our finding (2.4 µM), while Preston and colleagues [35] determined a high efficacy against *H. contortus* L3-L4 (IC_50_ 0.02–3 µM), the latter being in contrast with our results. Tolfenpyrad is a pesticide first authorized in Japan in 2002 under the trade name Hachi-hachi and later marketed as Tolfenpro^®^, a livestock acaricide. It was withdrawn for livestock use due to reports of eye inflammation as a side effect. Although tolfenpyrad is classified as “category 4: acute toxicity” according to the Globally Harmonized System of Classification and Labelling of Chemicals (GHS) and the European Chemicals Agency (ECHA), in silico analyses predict severe acute toxicity concerns. In addition, tolfenpyrad has been associated with severe erosion of the gastrointestinal mucosa in human poisoning cases, as reported in the clinical literature [36]. This effect is likely due to the inhibition of Complex I in the mitochondrial electron transfer chain. Plasma concentrations of 1.97 µg/mL (5.08 µM) were recorded in a poisoning case, thereby highlighting its high toxicity [37]. Additionally, Preston and colleagues demonstrated its ability to reduce respiratory rate in the rat hepatoma cell line (FAO) and estimated a maximum tolerated dose (MTD) of 1 mg/kg in mice [38]. These findings align with our toxicity results obtained in murine enteroids, thus warning about the oral use of this compound against GINs [39,40,41,42].

Octenidine is a synthetic disinfectant widely used for treating skin and mucosal lesions, and is particularly effective against Staphylococcus epidermidis, Escherichia coli, and Candida albicans. In vitro studies showed that concentrations below 1.5 μM led to a microbial reduction of more than 99% within 15 min. In vivo, octenidine demonstrates significant antimicrobial activity on cynomolgus monkey skin and effectively reduces dental plaque when used as a mouthwash. It has been investigated for skin disinfection in pediatric ICUs, treatment of upper respiratory tract infections, and prevention of oral infections [40]. Clinical trials have evaluated its efficacy and safety in treating conditions such as ringworm, Candida spp. infections, and infected eczema [42]. Recently, Elfawall and colleagues identified octenidine as a potential anthelmintic in a large-scale screening of 30,000 compounds against *Ancylostoma ceylanicum* and *Trichuris muris* [33], underscoring the relevance of phenotypic platforms in anthelmintic drug discovery. Predictive pharmacokinetics suggest that octenidine’s high molecular weight and lipophilicity contribute to its gastroenteric absorbability, which may be beneficial for intraluminal parasites [39,40,41,42]. Octenidine is classified as “category 4: acute toxicity”, indicating low toxicity. However, ADMETsar 2.0 analysis suggests that it may bind to the estrogen receptor and pose risks of hepatotoxicity, mitochondrial toxicity, and reproductive toxicity.

Chalcone and trans-chalcone are natural flavonoids primarily derived from *Glycyrrhiza inflata* and *Aronia melanocarpa*, respectively, and exhibit a wide range of biological and pharmacological activities. Chalcone derivatives are known for their anti-inflammatory, antioxidant, antibacterial, anticancer and antiparasitic effects. Trans-chalcone, a precursor of flavonoids, acts as a potent inhibitor of fatty acid synthase (FAS) and α-amylase, with notable antifungal and anticancer properties, including the ability to induce cell-cycle arrest and apoptosis in breast cancer cells [40]. Turani and colleagues [43] identified chalcone and its derivatives as nematocidal compounds against *C. elegans*, with an EC_50_ of 52 ± 9 µM. Notably, this result is very similar to the EC_50_ values obtained in the present work (52.4 ± 1.4 µM). These authors demonstrated that levamisole-sensitive acetylcholine receptors (L-AChR), the target of the known anthelmintic, were not involved in chalcone toxicity, although the mode of action remained unidentified [43]. Chalcone and trans-chalcone are classified as “category 4: acute toxicity”, indicating low toxicity. In silico analysis suggested similar pharmacokinetic and toxicological profiles for both isoforms. They exhibit high gastrointestinal absorption and can cross the blood–brain barrier. ADMET predictions indicate no hepatotoxicity or nephrotoxicity and a negative AMES test result, suggesting low mutagenic potential. Lee and colleagues [44] tested chalcone and its derivatives on zebrafish embryos, finding that derivatives, but not chalcone, induced muscular tissue malformations. Overall, chalcone and trans-chalcone appear promising due to their favorable safety profiles and high solubility, which facilitates the development of water-based formulations [39,40,41,42].

Despite these promising results, our study has limitations that require several considerations. First, the mechanism of action of the compounds identified remains elusive, with minimal clues available in the literature. Given the current gaps in our understanding of helminth biology, elucidating the precise mechanism of action will be challenging, althouth it is an essential step to optimize these compounds and predict potential resistance mechanisms. Moreover, while our in vitro assays using *C. elegans*, HepG2 spheroids, and mouse intestinal organoids have provided valuable insights into the efficacy and acute toxicity of these compounds, their pharmacokinetic and pharmacodynamic profiles have not yet been explored. For instance, although chalcone and trans-chalcone exhibited a favorable safety profile in our acute toxicity tests, further in vivo studies are necessary to assess their bioavailability, therapeutic indices, and potential side effects. Additionally, our study did not address the effects of chronic exposure or repeated administration. Given that anthelmintic treatments are often administered over prolonged periods, the long-term impact on both the host and the parasite remains uncertain, and cumulative toxicities may emerge over sustained use. The potential for off-target effects and interactions with host tissues also remains to be evaluated. Another limitation is the lack of investigation into the environmental fate of these compounds and their metabolites. With the prospect of mass use of any new anthelmintic drug, it is crucial to assess the risk of environmental contamination and its impact on non-target organisms and overall ecosystem health.

Overall, while our findings represent a significant step forward in the discovery of novel anthelmintics, these limitations underscore the need for comprehensive follow-up studies to fully characterize the safety, efficacy, and environmental impact of these compounds prior to clinical application.

## 4. Materials and Methods

### 4.1. Library and Compounds Management

The study was performed using five commercial compound libraries comprising a total of 2228 compounds: Anti-Infection Compound Library (MCE^®^ MedChemExpress, Monmouth Junction, NJ, USA, HY-L002), flavonoids library (MCE^®^ MedChemExpress, HY-L068), alkaloids library (MCE^®^ MedChemExpress, HY-L071), terpenoids library (MCE^®^ MedChemExpress, HY-N1504) and the FDA-approved Traditional Chinese Medicine library (MCE^®^ MedChemExpress, HY-L163). Hygromycin B (Corning, One Riverfront, NY, USA, 30-240-CR) and Propranolol (Ehrenstorfer, Teddington, Middlesex, UK, DRE-C16501000) were sourced from Fisher Scientific, while the rest of compounds were provided by MCE^®^ MedChemExpress with the same reference in the library list. Compounds were stored and handled following the manufacturer’s instructions. When storage at different temperatures was available, the lowest storage temperatures were used. Dissolved solutions of the compounds were freshly prepared and used within 1 month.

### 4.2. High Throughput Screening on C. elegans

*C. elegans* Bristol N2 strains, obtained from the Caenorhabditis Genetics Center (CGC; Minneapolis, MN, USA), were grown at 20 °C on Nematode Growth Medium (NGM) agar plates seeded with the OP50 strain of *Escherichia coli*, following standard procedures.

*C. elegans* fourth instar larvae (L4) were harvested by adding 6 mL of M9 buffer to the Petri dish containing the worms and transferring them to a 15 mL conical tube. This step was repeated with an additional 5 mL of M9 buffer, and the total volume was adjusted to 10 mL if necessary. Throughout handling, the worms were gently shaken on an orbital platform to minimize stress and prevent mortality. A 10 µL aliquot of the worm suspension was briefly incubated at −20 °C for 30 s, and live worms were then counted. The worm concentration was calculated, and 50 worms in 45 µL of M9 buffer were dispensed into each well of a non-treated 96-well U-bottom plate. Negative control wells contained 1% DMSO while the positive control contained 100 µM ivermectin. For the SS test, 45 µL of each compound dissolved in M9 buffer was added to achieve a final concentration of 110 µM in each well. For the DR test, a serial dilution of the testing compounds was prepared before addition to the plates. The plates were sealed with Parafilm and incubated at 20 °C, and readouts were taken at 0 h and after 24 h. The motility of worms was registered in WMicroTracker ONE^®^ instrument (Phylumtech, Santa Fe, Argentina, Hardware Version: WMTK09-R01/V1.4-R01, Software Version: WMTK 3.1) with the 1_Threadshold AVG modality. Cutoff values were set as ≥70% inhibition in the SS test and EC_50_ < 60 µM for the DR test.

### 4.3. Tests of Susceptibility on Parasitic Helminths

Eggs and third instar larvae (L3) of *H. contortus* from a field strain and a resistant strain of *T. circumcincta* were used in the current trials. These parasitic forms were obtained from fresh feces of Merino and Churra sheep experimentally infected at the experimental farm of the Instituto de Ganadería de Montaña (IGM, CSIC, Gruyeros, León, Spain) and purified according to previously described methods. Briefly, eggs were taken from fresh feces, washed, centrifuged and floated with a saturated sodium chloride solution, then concentrated, counted and used in the assays. L3 were obtained by culturing feces at 25 °C for 13 d. L3 larvae were ensheathed in 0.5% sodium hypochlorite solution for 10 min at room temperature and washed to avoid excess salt. After washing, they were concentrated and counted for use.

To test for anthelmintic efficacy, procedures from previous studies were followed [45,46]. First, a SS screening at 50 µM was performed using the Egg-Hatching Test (EHT) on the susceptible isolate of *H. contortus*. EHT was performed in 24-well microplates, using 1000 µL aliquots containing about 150 fresh parasite eggs in each well. To perform the initial screening, 10 µL of a 10 mM stock solution of each testing compound and 1990 µL of distilled water were added to each well to reach a final volume of 2000 µL and a final concentration of 50 µM. After 48 h of incubation at 25 °C, the number of L1 and eggs present per well was counted using an inverted microscope to determine the percentage of hatched eggs:

$$\%\;Egg\;hatching=\left(\frac{number\;of\;L1}{number\;of\;L1+number\;of\;eggs}\right)\times 100$$

The ovicidal effect was expressed as the percentage of egg hatching inhibition using the following formula:

$$\%\;Egg\;hatching\;inhibition=\left(1-\frac{\%\;egg\;hatch\;treated}{\%\;egg\;hatch\;control}\right)\times 100$$

The EC_50_ of the compounds was calculated using at least 8 serial dilutions 1:2, ranging from 50 µM to 0.39 µM in 9 replicates. EC_50_ values were determined by plotting the percentage of hatched eggs against the concentration of each compound, using the nonlinear fit analysis provided by the SigmaPlot^®^ 10.1. As controls, Thiabendazole at 0.1 µg/mL and DMSO at 0.5% (*v*/*v*) were used.

The viability of *H. contortus* larvae was assessed by the larval migration inhibition test (LMIT) according to previously described methods [46,47]. Briefly, 700 µL aliquots containing 500–600 L3 were prepared for each compound. To these larvae, 693 µL of distilled water and 7 µL of the test compound from a 10 mM stock solution were added to reach a final concentration of 50 µM in a volume of 1400 µL. After a 24 h incubation at 28 °C, the compound was removed by three washes, leaving a final volume of 1400 µL. After the washes, three 400 µL aliquots were taken from each tube and transferred to a 96-well plate with a MultiScreen mesh filter. The plates were incubated for a further 24 h at 28 °C to allow the motile L3 to pass through the 20 µm mesh for counting. Then, the number of larvae present in each well was counted to determine percentage of larval migration for each compound:

$$\%\;Larval\;migration=\left(\frac{number\;of\;larvae\;migrating\;in\;compound\;wells}{number\;of\;larvae\;migrating\;in\;negative\;control}\right)\times 100$$

The number of larvae present in each well was then counted to determine the percentage of larval migration for each compound:

% Larval migration inhibition=100−% Larval migration

For the larvae, ivermectin at 8.75 µg/mL and DMSO 0.5% (*v*/*v*) were used as controls and at least 12 replicates were performed. Compounds that showed effects above 90% were selected for the determination of their half-maximal effective concentration (EC_50_). The EC_50_ of the compounds was calculated using at least 8 serial dilutions 1:2, ranging from 50 µM to 0.39 µM. EC_50_ were determined by plotting the percentage of viability of the L1 against the concentration of each compound, using the nonlinear fit analysis provided by the SigmaPlot^®^ 10.1.

### 4.4. Spheroids Toxicity Assay

HepG2 cells were cultured as a monolayer under standard conditions, using DMEM/F12 medium supplemented with L-glutamine and 15 mM HEPES buffer (pH 7.2) supplemented with 10% fetal bovine serum (FBS) and antibiotic mixture (100 U/mL penicillin and 100 µg/mL streptomycin) at 37 °C and 5% CO_2_, according to previous published protocols.

Cells were detached from the culture flask using 0.25% trypsin solution and then centrifuged at 500× *g* for 6.5 min. After centrifugation, the supernatant was discarded, and the pellet was resuspended in culture medium. Cells were passed gently through a G27 needle several times and then counted. Cells were dispensed into a tissue treated Nunclon™ Sphera™ 96-well U-bottom plate (Thermo Scientific™, Waltham, MA, USA, 174925) in a final volume of 50 µL to a total of 2500 cells/well. In addition, another 50 µL of medium supplemented with Rat Tail Collagen I (Gibco™, Thermo Scientific™, Waltham, MA, USA, A1048301) was dispensed, to achieve a final concentration in a plate of 3 µg/mL for each well. The plate was centrifuged at 500× *g* for 1 min.

On Day 4, 100 µL of a serial dilution of the testing compounds were added to each well. For controls, 0.03% H_2_O_2_ (*v*/*v*) as positive control and 0.2% DMSO (*v*/*v*) as negative control were used.

To determine the viability of the spheroids to the testing compounds, a 20% of alamarBlue™ HS reagent (Invitrogen™, Thermo Fisher Scientific™, Waltham, MA, USA) was added on Day 7 and the results were read 24 h later using a Varioskan™ LUX (Thermo Scientific, USA, Instrument software ver. 1.00.37, Skanlt software ver. 5.0.0.42). All assays were conducted in duplicate across at least three independent experiments.

### 4.5. Mouse Intestinal Organoids’ Tolerance Assay

To determine the in vitro tolerance to the compounds identified with anthelmintic properties in *C. elegans*, mouse intestinal organoids were cultured following an adapted protocol based on the Intestinal Epithelial Organoid Culture with an IntestiCult™ Organoid Growth Medium (Mouse) technical bulletin (https://cdn.stemcell.com/media/files/techbulletin/TB28223-Intestinal_Epithelial_Organoid_Culture_with_IntestiCult_Organoid_Growth_Medium_(Mouse).pdf?_ga=1.255806536.1701332796.1451577632, accessed on 6 September 2022) [48]. To obtain intestine stem cells, C57BL/6 mice were used. Briefly, after humane euthanasia, a section of mouse duodenum and jejunum was removed, rinsed in ice-cold PBS, and cleared of contents. Tissue was cut into segments that were washed 15-20 times with PBS and incubated in Gentle Cell Dissociation Reagent (GCDR, Stemcell Technologies™, Vancouver, BC, Canada) for 20 min. The segments were resuspended in PBS containing 0.1% BSA and pipetted 3 times. The supernatant was filtered through a 70 µm strainer, collected as “Fraction 1”, and the process was repeated 4 times. Fractions were centrifuged at 300× *g*, 5 min at 4 °C, resuspended in PBS containing 0.1% BSA, centrifuged again at 300× *g* for 3 min and resuspended in DMEM/F12. The selected fraction was centrifuged at 200× *g*, 5 min, 4 °C, and the pellet resuspended in a 1:1 mixture of Geltrex™ GFR LDEV-free (Gibco, Fisher Scientific, Waltham, MA, USA) and IntestiCult™ Mouse Organoid Growth Medium (IntestiCult™ mouse OGM, Stemcell Technologies™, Vancouver, BC, Canada). A total of 50 µL of this suspension was pipetted into pre-warmed 24-well plates to form domes. After incubation at 37 °C for 10 min to allow for matrix polymerization, 500 µL of IntestiCult™ mouse OGM were added. Plates were maintained at 37 °C with 5% CO_2_, and the medium was refreshed every 2–3 days. All animal procedures were performed in compliance with Spanish and EU legislation (RD 53/2013 and 2010/63/EU) and were approved by the Ethics Committee of the University of León (Project license OEBA-ULE-007-2019).

Organoids were passaged every 7 d. Medium was removed, and domes were incubated with 800 µL of GCDR for 1 min, followed by pipetting to disrupt the matrix. The suspension was transferred to a 15 mL tube, and the wells were washed with another 800 µL of reagent. After a further 10 min of incubation on a rocking platform, the suspension was centrifuged at 300× *g* for 5 min. The pellet was resuspended in 5–10 mL of DMEM/F12 with L-glutamine and HEPES and centrifuged again at 200× *g* for 5 min. Finally, the pellet was resuspended and plated as in the above paragraph.

The 384-well format assay was adapted from Du and coworkers [23] with several modifications. After discarding the medium, organoids were harvested from the matrix for passaging and the pellet was resuspended in 3.6 mL of a 1:1 mixture of Geltrex™ GFR LDEV-free and IntestiCult™ mouse OGM. Using a frozen pipette dispenser, 8 µL of the organoid suspension was dispensed into pre-frozen 384-well plates, followed by manual shaking to distribute the matrix. Plates were incubated at 37 °C for 10 min and 32 µL of IntestiCult™ mouse OGM was added to each well, and 100 µL of sterile water was added to the perimeter wells. On Day 4, compounds dissolved in IntestiCult™ mouse OGM were added in the quantity of 10 µL/well. Positive (0.15% H_2_O_2_ *v*/*v*) and negative (0.2% DMSO *v*/*v*) controls were added. On Day 7, 5 µL of alamarBlue™ HS (Invitrogen™, Thermo Fisher Scientific™, USA) were added to each well, and after 3 h, fluorescence was measured using a Varioskan™ LUX microplate reader (Thermo Scientific, USA, Instrument software ver. 1.00.37, Skanlt software ver. 5.0.0.42). All assays were conducted in triplicate across at least three independent experiments.

### 4.6. In Silico Analysis of the Hits

The SMILES representations of the compounds were input into SwissADME and ADMETsar 2.0 software for in silico evaluation [49,50,51]. The main results from these analyses were then examined to assess key pharmacokinetic and toxicological properties.

SwissADME [49] employs OpenBabel to calculate physicochemical properties such as molecular weight (MW), molecular refractivity (MR), and polar surface area (PSA). Lipophilicity is determined using five different predictive models (XLOGP3, WLOGP, MLOGP, SILICOS-IT and iLOGP), and a consensus value is generated by taking the arithmetic mean of these values. Water solubility is predicted using three topological methods, ESOL, and a method developed by SILICOS-IT. The software also uses the BOILED-Egg model to predict passive human gastrointestinal absorption (HIA) and blood–brain barrier (BBB) permeation, using a graphical classification based on lipophilicity and polarity. In addition, support vector machine (SVM) algorithms are applied to predict whether a molecule is an inhibitor of various cytochrome P450 (CYP) isoenzymes (CYP1A2, CYP2C19, CYP2C9, CYP2D6 and CYP3A4).

The admetSAR 2.0 tool [50,51] uses a range of machine learning algorithms to build predictive models for ADMET properties. The models are trained using data from sources such as DrugBank, ChEMBL, CPDB, peer-reviewed papers and high-throughput screening projects. Molecular structures are represented using molecular fingerprints such as MACCS, Morgan and AtomPairs with RDKit. Machine learning algorithms, such as random forest (RF), support vector machine (SVM) and k-nearest neighbors (kNN), are used to build the classification models, and a 5-fold cross-validation is employed to optimize their hyper-parameters. Multi-label methods like Binary Relevance, Classifier Chains and Label Powerset are used for predicting endocrine disrupting properties. The predictive performance of the models is evaluated using metrics like AUC for classification models, and R^2^ for regression models. The models also have an applicability domain (AD), which is defined upon physicochemical and topological properties. ADMETopt, a lead optimization module, uses a scaffold hopping approach that replaces molecular scaffolds based on the Tanimoto coefficient of scaffold descriptors. This process filters new molecules based on the predicted ADMET properties.

### 4.7. Statistical Analysis

To validate the reliability of the assays, several statistical evaluations were performed. The Z′-factor was calculated along with measures of signal-to-background ratio, signal-to-noise ratio, signal window, and assay variability ratio, following the methodology outlined by Iversen and co-workers [52,53,54,55].

For the *C. elegans* assay, the Z′-factor test was conducted in a 2 × 3 format, using ivermectin at a final concentration of 100 µM to represent the minimum signal and 1% DMSO (*v*/*v*) as the maximum signal. For the HepG2 spheroids assay, the Z′-factor test had a 2 × 3 format with 0.03% (*v*/*v*) hydrogen peroxide to represent the minimum signal and 0.2% DMSO (*v*/*v*) as the maximum signal. For the mouse intestinal organoids assay, the Z′-factor test had a 2 × 2 format with 0.02% (*v*/*v*) hydrogen peroxide to represent the minimum signal and 0.2% DMSO (*v*/*v*) as the maximum signal.

For the *C. elegans* SS assay, data points were collected from two independent experiments, for a total of four data points per compound. Positive and negative controls were cleaned by removing outliers, defined as:

Value<Q1−1.5×IQR

Value>Q3+1.5×IQR

Q indicates the corresponding quartile and IQR is the interquartile range between Q3 and Q1. Data from the same experiment were averaged, and pre-hits were selected if the 4 data points average demonstrated inhibition greater than 70% relative to their respective negative controls.

For the *C. elegans* DR assay and toxicological analysis, a minimum of six data points were collected from at least three independent experiments. Each data point was normalized to its respective negative control and outlier removal followed the same method as in the SS assay.

The data were then exported to SigmaPlot^®^ 10.0 (Grafiti LLC, USA, built 10.0.0.54), where standard curve analysis was performed using the logarithmic normalization of compound concentrations. Dose–response curves were deemed of sufficient quality if they had an R-value greater than 0.90 and a standard error of EC_50_ values less than 10%.

Unless otherwise stated, statistical analysis and graphic elaborations were realized using Microsoft^®^ Office Professional Plus 2019.

## 5. Conclusions

This study demonstrates the potential of the *C. elegans* motility inhibition platform under HTS conditions for identifying novel anthelmintic compounds. The platform successfully identified candidates with distinct safety profiles, toxicity levels, and selective activity against parasitic nematodes, supporting their further development as therapeutic agents. Among the identified compounds, the natural flavonoids chalcone and trans-chalcone exhibited robust anthelmintic efficacy, minimal toxicity, and good intestinal tolerability, making them promising candidates for further investigation. Conversely, despite their strong anthelmintic effect, the synthetic drugs tolfenpyrad and octenidine exhibited high toxicity in intestinal organoids. This underscores the necessity for alternative delivery strategies or specific applications targeting external or non-mucosal parasitic infections. Future research should focus on optimizing the pharmacokinetics and therapeutic indices of these compounds, particularly chalcone derivatives, to fully exploit their therapeutic potential.

## Figures and Tables

**Table 1:** The effectiveness of the 32 compounds that reduced motility by more than 70% at 110 μM after 0 h and 24 h of exposure in the SS experiments and EC_50_ values obtained in DR assays. The results represent the mean ± SD of four experiments.

Compound	Collection	CAS Number	Inhibition (%) 110 μM (0 h)	Inhibition (%) 110 μM (24 h)	EC_50_ (μM) at 24 h
Avermectin B1	Anti-Infection	71751-41-2	81 ± 1	42 ± 1	NT **
Chloroxylenol		88-04-0	100 ± 0	100 ± 0	>60 *
Eprinomectin		123997-26-2	91 ± 3	92 ± 3	NT **
Ethacridine lactate		1837-57-6	66 ± 26	87 ± 6	50.1 ± 1.0
Ethacridine lactate monohydrate		6402-23-9	73 ± 17	89 ± 4	28.2 ± 1.0
Fidaxomicin		873857-62-6	82 ± 7	89 ± 1	>60 *
Hygromycin B		31282-04-9	67 ± 28	89 ± 5	>60 *
Ivermectin		70288-86-7	98 ± 1	85 ± 14	NT **
Levamisole		14769-73-4	87 ± 10	61 ± 3	NT **
Milbemycin		51596-10-2	100 ± 0	86 ± 14	NT **
Moxidectin		113507-06-5	99 ± 1	85 ± 13	NT **
Nitroxoline		4008-48-4	52 ± 32	88 ± 2	>60 *
Octenidine		70775-75-6	84 ± 1	96 ± 5	6.9 ± 0.5
Oxyclozanide		2277-92-1	10 ± 9	93 ± 5	NT **
Pyrantel pamoate		22204-24-6	94 ± 0	18 ± 18	NT **
Pyrantel tartrate		33401-94-4	71 ± 1	26 ± 1	NT **
Ribavirin		36791-04-5	91 ± 5	76 ± 11	>60 *
Robenidine		25875-51-8	93 ± 5	81 ± 20	32.3 ± 2.8
Salinomycin		53003-10-4	81 ± 9	94 ± 1	55.3 ± 1.4
Spiramycin		8025-81-8	43 ± 42	81 ± 9	>60 *
Sulfaquinoxaline		59-40-5	87 ± 1	67 ± 6	>60 *
Tetramisole		5086-74-8	78 ± 2	82 ± 3	NT **
Tolfenpyrad		129558-76-5	80 ± 2	98 ± 2	2.4 ± 0.2
Kuwanon G	Flavonoids	75629-19-5	0 ± 0	74 ± 5	34.7 ± 1.6
Chalcone		614-47-1	30 ± 30	85 ± 15	52.4 ± 1.4
Trans-chalcone		614-47-1	16 ± 17	95 ± 5	24.9 ± 1.8
Polygodial	Terpenoid	6754-20-7	0 ± 0	93 ± 4	33.2 ± 6.4
3,29-dibenzoyl rarounitriol		873001-54-8	40 ± 22	71 ± 19	45.4 ± 5.1
Triptolide		38748-32-2	15 ± 0	72 ± 1	>60 *
Dioscin	Chinese	19057-60-4	12 ± 1	79 ± 2	37.8 ± 1.3
Propranolol		318-98-9	84 ± 0	35 ± 0	>60 *
Resveratrol		501-36-0	82 ± 8	7 ± 7	27.7 ± 1.5

* These compounds were discarded for further analyses in parasitic helminths. ** NT: not tested in DR experiments, since they correspond to compounds with known anthelmintic activity.

**Table 2:** The effectiveness (EHI and LMIT) of the 13 compounds identified in the *C. elegans* motility inhibition assay (EC_50_ values below 60 µM) on a susceptible strain of *H. contortus* and a resistant strain of *T. circumcincta*. The results represent the averages and SD of a minimum 9 replicates for EHI and 12 for LMIT.

Compound	Collection	*H. contortus* EHI (%)	*H. contortus* LMIT (%)	*T. circumcincta* EHI (%)	*T. circumcincta* LMIT (%)
		**Susceptible Strain**		**Resistant Strain**	
Ethacridine lactate	Anti-Infection	55.8 ± 5.1	4.9 ± 2.2	4.0 ± 1.5	NE *
Ethacridine lactate monohydrate		54.8 ± 11.7	0.0	0.0	0.0 *
Octenidine		99.5 ± 0.8	98.3 ± 0.3	99.5 ± 0.9	94.8 ± 4.3
Robenidine		59.7 ± 0.7	3.1 ± 6.9	12.5 ± 0.70.0	NE *
Salinomycin		2.8 ± 5.1	0.0	NE	NE *
Tolfenpyrad		96.0 ± 2.9	9.0 ± 6.3	99.7 ± 0.5	NE
Kuwanon G	Flavonoids	9.4 ± 1.8	0.0	NE	NE *
Chalcone		99.4 ± 0.1	45.7 ± 4.5	100.0 ± 0.0	4.7 ± 1.5
Trans-chalcone		99.5 ± 0.7	20.3 ± 7.5	99.9 ± 0.2	NE
Polygodial	Terpenoid	0.0	0.0	NE	NE *
3,29-dibenzoyl rarounitriol		0.0	0.0	0.0	0.0 *
Dioscin	FDA Chinese	NE	0.0	NE	0.0 *
Resveratrol		0.0	0.0	NE	NE *
Thiabendazole	Controls	100.0 ± 0.0	-	100.0 ± 0.0	-
Ivermectine		-	100.0 ± 0.0	-	100.0 ± 0.0

NE: Not Effective at 100 μM: * These compounds were discarded for toxicological analyses.

**Table 3:** Efficacy (EC_50_) and cytotoxicity (CC_50_) in both HepG2 liver spheroids and mouse enteroids of the four compounds that reduced egg hatching by more than 90% at a single dose of 100 µM in the susceptible strain of *H. contortus* and the resistant strain of *T. circumcincta*, and their corresponding selective index (SI). Four technical replicates were performed for toxicological models and nine for the ovicidal assay.

	Ovicidal Effect EC_50_ (μM)		Citotoxicity CC_50_ (μM)		Selective Index (SI) CC_50_/EC_50_			
Compound	*H. c*	*T. c*	Sph	Ent	Sph/*H. c*	Ent/*H. c*	Sph/*T. c*	Ent/*T. c*
Octenidine	2.1 *±* 0.1	15.1 *±* 4.8	54.1 *±* 2.8	14.2 *±* 0.4	25.8	6.8	3.6	0.9
Tolfenpyrad	1.5 *±* 0.1	0.2 *±* 0.3	>50	<1	>33.3	<0.7	250	<5
Chalcone	3.7 *±* 0.1	9.8 *±* 0.3	>90	>50	>24.3	>13.5	>9.2	>5.1
Trans-chalcone	3.1 *±* 0.2	4.9 *±* 0.2	>90	>50	>29.0	>16.1	>18.4	>10.2
Thiabendazole	0.28 ± 0.01	1.53 ± 0.06	-	-	-	-	-	-

Ent (mouse enteroid); *H. c* (*H. contortus*); Sph (HepG2 liver spheroids); *T. c* (*T. circumcincta*).

**Table 4:** Principal values of ADMET properties calculated with SwissADME.

Compound	MW [g/mol]	Num. Rotable Bonds	Num. H Acceptor	Num. H Donor	Consensus LogP o/w	LogS ESOL *	GI Abs	BBB Pen	CYP Inh
Octenidine	623.83 **	25	2	0	8.12	−10.20 **	Low	No	No
Tolfenpyrad	383.87	7	3	1	4.23	−5.27	High	Yes	Yes
Chalcone	208.26	3	1	0	3.29	−3.43	High	Yes	Yes
Trans-chalcone	208.26	3	1	0	3.29	−3.43	High	Yes	Yes

* Estimated SOLubility: <−10 insoluble <−6 poorly soluble <−4 moderately soluble <−2 very soluble <0 highly soluble. ** violation of Lipinski rule. BBB perm (blood–brain barrier penetration); CYP inh (inhibition of cytochrome P450 enzymes); GI abs (gastrointestinal absorption).

## Data Availability

Raw data available in Zenodo repository: https://doi.org/10.5281/zenodo.14795234 (accessed on 11 February 2025).

## Supplementary Materials

The following supporting information can be downloaded at: https://www.mdpi.com/article/10.3390/ijms26041595/s1.

## Abbreviations

The following abbreviations are used in this manuscript:

AChR	Acetylcholine receptor
ADMET	Absorption, distribution, metabolism, excretion, toxicity
BBB	Blood–brain barrier
CC_50_	Cytotoxic concentration 50%
CYP	Cytochrome P450
DR	Dose response
EC_50_	Effective dose 50%
EHA	Egg-hatching assay
EHI	Egg-hatching inhibition
FAS	Fatty acid synthetase
GI	Gastrointestinal tract
GINs	Gastrointestinal nematodes
HTS	High-throughput screening
LMI	Larval migration inhibition
LMIT	Larval migration inhibition test
MTD	Maximum tolerated dose
SS	Single-slot
2D	Two-dimensional
3D	Three-dimensional
