# Supplementary material for: High-Throughput Screening of Five Compound Libraries for Anthelmintic Activity and Toxicity Leads to the Discovery of Two Flavonoid Compounds
Source: Int J Mol Sci. 2025 Feb 13;26(4):1595. doi: 10.3390/ijms26041595 (PMC11855827; doi:10.3390/ijms26041595)
Supplement: Supplementary file 1 [file ijms-26-01595-s001.zip › ijms-3458730-supplementary.pdf]

Supporting Information

High-Throughput Screening of Five Compound Libraries for Anthelmintic Activity and Toxicity Leads to the Discovery of Two Flavonoid Compounds

Giulio Galli [1], Marta Ruiz-Somacarrera [2], Laura González del Palacio [2], Estela Melcón-Fernández [1], Rubén González-Pérez [1], Carlos García-Estrada [1,3], María Martínez-Valladares [2,*] and Rafael Balaña-Fouce [1,3*]

[1] Departamento de Ciencias Biomédicas, Facultad de Veterinaria, Universidad de León, Campus de Vegazana s/n, 24071 León, Spain
[2] Departamento Sanidad Animal, Instituto de Ganadería de Montaña, CSIC-Universidad de León, Grulleros, 24346 León, Spain
[3] Instituto de Biomedicina (IBIOMED), Universidad de León, Campus de Vegazana s/n, 24071 León, Spain
* Correspondence: mmarva@csic.es (M.M.-V.); rbalf@unileon.es (R.B.-F.)

Table S1. Values obtained from the Z'-factor test and respective averages for the *C. elegans* assay. P1-6 indicate 6 independent experiments in 3 different days. Reference values: Z'>0.5, SW>2, AVR<0.5.

	P1	P2	P3	P4	P5	P6	**Average**
**Z'**	0.8	0.7	0.8	0.7	0.8	0.7	0.8
**S/B**	1386	168	149	17	80	28	305
**S/N**	10.2	1.7	1.0	0.4	0.9	0.4	2.4
**SW**	9.4	9.1	12.3	8.0	17.5	11.9	11.4
**AVR**	0.2	0.3	0.2	0.3	0.2	0.3	0.2

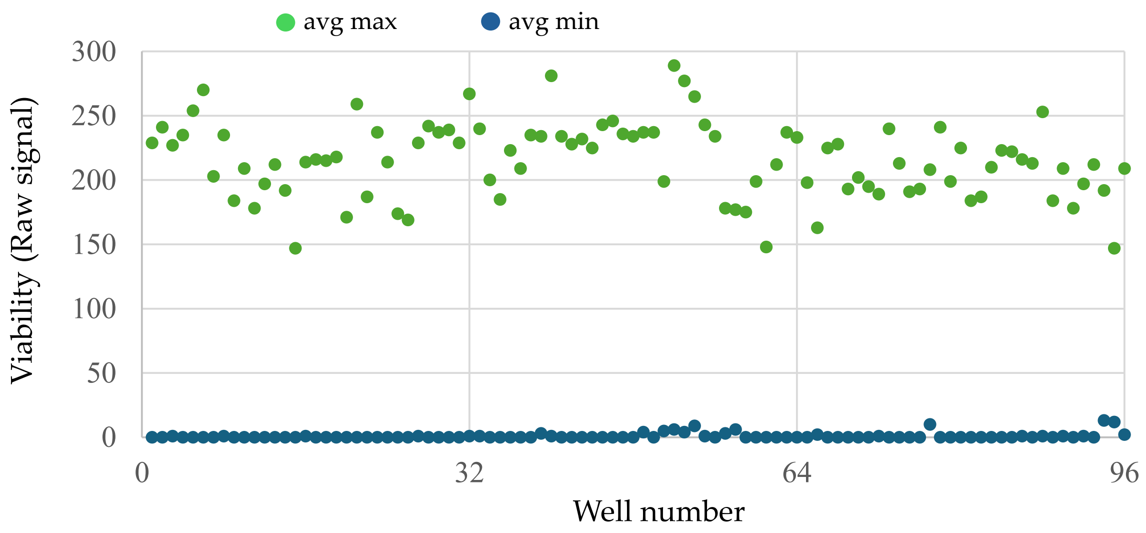

Figure S1. Raw signal per well recorded during Z′-factor test (averages) for the *C. elegans* assay.

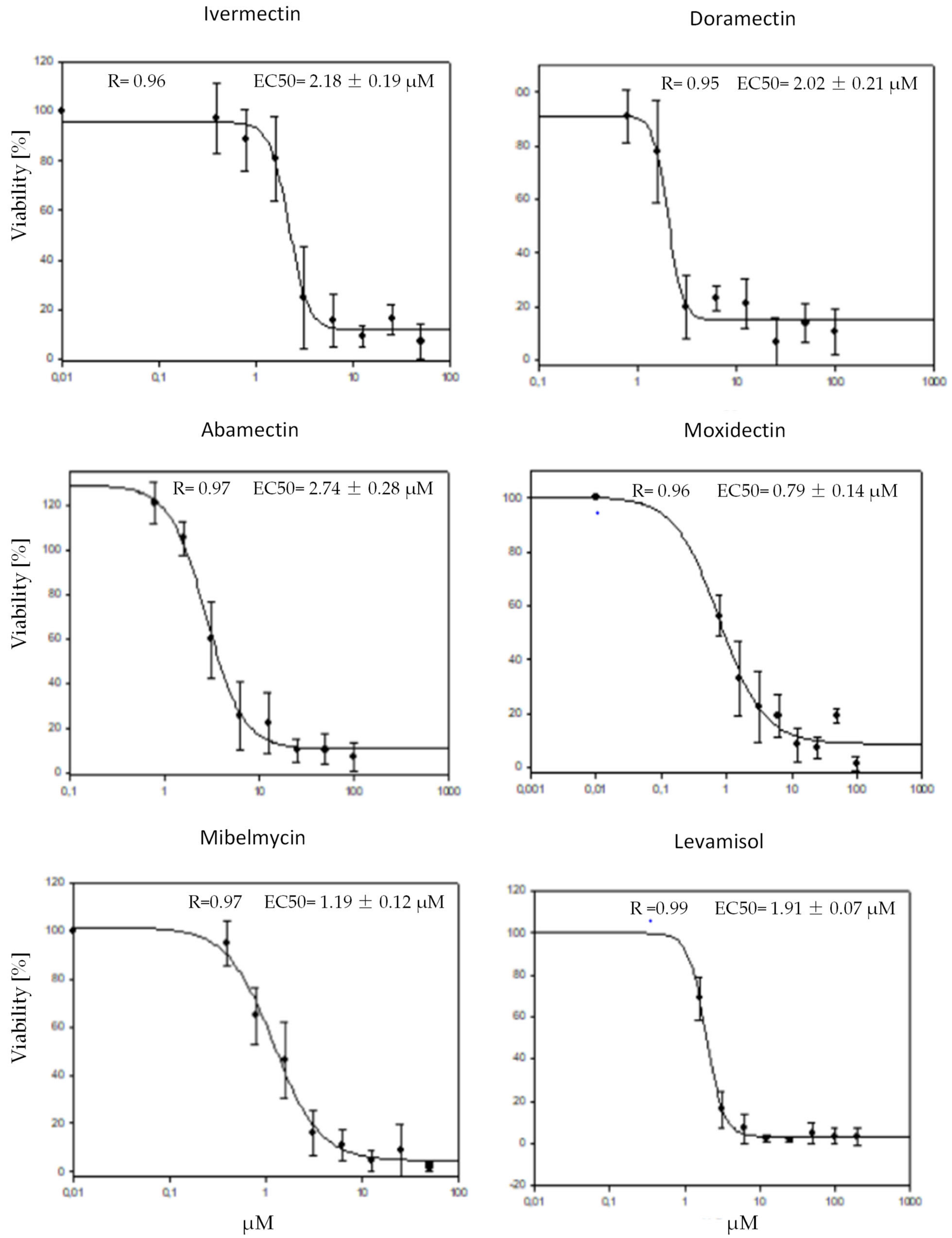

Figure S2. Dose-effect curves of the DR assay on *C. elegans* at 24 h using compounds with known anthelmintic properties. Plots were elaborated with SigmaPlot® 10.0. Drugs were tested at different concentrations ranging from 200 μM to 0.01 μM.

Table S2. List of 2,228 compounds tested in *C. elegans* SS assay and relative percentage of motility inhibition (averages of 4 data points) at 0 h and 24 h. All compounds were tested at 110 μM and supplied by MCE® MedChemExpress, USA.

Reference	Compound name	0 h	24 h
HY-B1751	Quinidine (15% dihydroquinidine)	36%	18%
HY-113262	8-Hydroxyguanosine	14%	4%
HY-N2352A	Laurolitsine (hydrochloride)	36%	5%
HY-N4261	Dehydronuciferine	6%	6%
HY-N0714A	Berbamine (dihydrochloride)	33%	11%
HY-B1167	Ajmaline	19%	6%
HY-N0288	Lycorine	34%	0%
HY-15122	Sinomenine	18%	3%
HY-N7263	Galanthamine N-Oxide	3%	4%
HY-B0979	Lobeline (hydrochloride)	26%	30%
HY-W002168	1,3-Dimethylpyrazole	6%	3%
HY-Y0061	Oxindole	14%	7%
HY-N0388	Gelsemine	0%	0%
HY-121936A	Yohimbic acid (hydrate)	16%	0%
HY-B0997	Hydroquinidine	29%	21%
HY-W001132	Indole	13%	9%
HY-113239	Hydroxycotinine	13%	0%
HY-N1974	Fuziline	38%	7%
HY-N0862	Harringtonine	25%	0%
HY-N0740	Jatrorrhizine (chloride)	33%	4%
HY-N0930B	Galegine (hydrochloride)	0%	0%
HY-N1372	(R)-Fangchinoline	44%	1%
HY-N6691	Veratridine	4%	0%
HY-Y0265	Isatin	25%	3%
HY-N1584A	Halofuginone (hydrobromide)	4%	0%
HY-N0478	Neoline	21%	1%
HY-W010195	2,6-Dimethylquinoline	0%	6%
HY-N1402	Oxysophoridine	18%	9%
HY-N1882	4,5-Dimethoxycanthin-6-one	0%	22%
HY-100971	Spiramide	49%	6%
HY-N0096	Rotundine	0%	4%
HY-N2322	Khasianine	19%	10%
HY-N0924A	(±)-Stylopine (hydrochloride)	0%	3%
HY-N0450	Sinapine (thiocyanate)	0%	0%
HY-113081	1-Methyladenosine	7%	0%
HY-N0471A	L-Hyoscyamine (sulfate)	18%	9%
HY-B1205	Atropine	3%	3%
HY-N0071	Crotonoside	0%	0%
HY-N6857	Armepavine	0%	12%
HY-N6638	Retrorsine	28%	9%
HY-N2410	N-trans-Feruloyltyramine	5%	16%

HY-17387	(-)-Huperzine A	0%	4%
HY-N6931	Usaramine	31%	21%
HY-16562A	Irinotecan (hydrochloride)	0%	11%
HY-N7401	Entadamide-A-β-D-glucopyranoside	24%	13%
HY-17024	Cyclopamine	0%	14%
HY-N0158	Oxymatrine	15%	11%
HY-N9404	6-Benzoylheteratisine	16%	23%
HY-N2021A	Phosphoramidon (Disodium)	19%	7%
HY-W091541	4-Hydroxy-1H-indole-3-carbaldehyde	23%	5%
HY-N0413	Hupehenine	0%	10%
HY-N0132A	Synephrine (hydrochloride)	0%	4%
HY-N0826	Corynoline	13%	0%
HY-15141	Staurosporine	15%	33%
HY-N0441	Neferine	25%	9%
HY-N4068	Glucoraphanin	12%	0%
HY-A0009	Galanthamine (hydrobromide)	2%	13%
HY-W012185	(-)-Sparteine	0%	4%
HY-N0849	Dictamine	0%	20%
HY-113440	5-Methoxytryptophol	25%	7%
HY-W018800	4(3H)-Quinazolinone	0%	0%
HY-N0275	(±)-10-Hydroxycamptothecin	0%	0%
HY-N7271	Solanidine	0%	0%
HY-N6607	Tryptanthrin	11%	0%
HY-W016887	H-Gly-Pro-OH	8%	4%
HY-N2043	Huperzine B	18%	8%
HY-N0674B	Dehydrocorydaline (hydroxyl)	2%	0%
HY-W042156	Aegeline	4%	6%
HY-135005	Biliverdin (hydrochloride)	0%	1%
HY-N0220	Dauricine	2%	9%
HY-N2625A	Harmalol (hydrochloride)	17%	2%
HY-N0329	Deltaline	0%	0%
HY-N6973	Boldine	0%	0%
HY-124124	N-Methylnicotinamide	3%	0%
HY-B1304A	(+)-Sparteine (sulfate pentahydrate)	0%	0%
HY-B0927	Hydrastine	0%	19%
HY-N0443	N-Methylcytisine	0%	0%
HY-129583	Lepidiline A	5%	14%
HY-N2064	Racanisodamine	11%	0%
HY-N0449	Nordihydrocapsaicin	0%	4%
HY-N2329	Piperlongumine	0%	0%
HY-N0586	Norisoboldine	0%	0%
HY-100807	Quinolinic acid	0%	0%
HY-N0117	Indirubin	5%	6%
HY-N2179	Hypaphorine	0%	0%

HY-W008566	Norharmane	0%	9%
HY-N0716B	Berberine (sulfate)	0%	0%
HY-111914A	Ferroheme	0%	0%
HY-N0173	Cinchonidine	0%	0%
HY-N0050	Allomatrine	0%	0%
HY-N2384	Febrifugine	0%	0%
HY-B0188A	Mianserin (hydrochloride)	0%	0%
HY-N2403	Dihydrolycorine	0%	0%
HY-15753A	Ellipticine (hydrochloride)	9%	11%
HY-125827	13-Methylberberine (chloride)	0%	0%
HY-N0903	Dihydrochelerythrine	0%	7%
HY-U00082	Tigloidin	0%	0%
HY-W007426	N-Methylbenzylamine	0%	4%
HY-W015818	2-Benzoxazolinone	24%	6%
HY-15122A	Sinomenine hydrochloride	0%	0%
HY-N5016	Guvacoline (hydrochloride)	0%	0%
HY-B1181A	Hydrastinine	1%	4%
HY-N2591	Isocorydine	0%	0%
HY-W001160	5-Hydroxyindole	28%	9%
HY-B0394	Atropine (sulfate monohydrate)	0%	17%
HY-B0809	Theophylline	21%	15%
HY-N2149	Tomatidine	6%	0%
HY-13295	Vinpocetine	0%	14%
HY-131413	O-Desmethyl Galanthamine	4%	5%
HY-N1343	Robustine	16%	7%
HY-126562	Piperlonguminine	34%	22%
HY-N2392	Kukoamine A	0%	32%
HY-N0735	Phellodendrine (chloride)	25%	7%
HY-W012956	2-Acetylpyrrole	26%	18%
HY-100385	Brevianamide F	31%	18%
HY-B1180	Vinburnine	0%	15%
HY-N0738	Stachydrine hydrochloride	15%	26%
HY-N2081	Skimmianine	0%	15%
HY-N0334A	(+)-Magnoflorine (iodide)	10%	8%
HY-N7031	(±)-Vasicine	3%	31%
HY-N2093	Vicine	0%	0%
HY-101392	Harmane	0%	6%
HY-22385	Salsolidine	0%	0%
HY-N0069	Solamargine	0%	0%
HY-N7674	Angoline	0%	0%
HY-13780	Vinblastine (sulfate)	0%	0%
HY-42034	Hydroquinine	0%	0%
HY-B0739	Citicoline	0%	0%
HY-N0766	Isorhynchophylline	0%	0%

HY-W007140	2-Hydroxymethyl-5-hydroxypyridine	0%	0%
HY-W016784	Indole-3-acetamide	0%	0%
HY-N0070	Solasonine	0%	0%
HY-N0166	Gramine	0%	0%
HY-N0838	Cephalotaxine	0%	0%
HY-N1100	Vasicinone	0%	0%
HY-N6619A	Lycoramine	0%	0%
HY-N2255	Crebanine	0%	4%
HY-W005963	Methyl 5-hydroxypyridine-2-carboxylate	7%	1%
HY-N2369	Chelidonine	0%	0%
HY-100809	Guvacine hydrochloride	0%	0%
HY-N0103A	Sophocarpine (monohydrate)	0%	0%
HY-N5009	Thermopsine	0%	2%
HY-17578	Pneumocandin B0	0%	0%
HY-N0252	Catharanthine	0%	0%
HY-13704	SN-38	34%	3%
HY-N0252A	Catharanthine (Tartrate)	0%	12%
HY-N3182	N-Methylnuciferine	0%	0%
HY-N0298	Stachydrine	0%	0%
HY-N0901A	Corynoxine B	5%	20%
HY-113432	Nudifloramide	0%	0%
HY-W047478	3-Methylcarbazole	0%	29%
HY-N4317	Ethoxysanguinarine	0%	31%
HY-N1282	Seneciphylline	0%	1%
HY-N0144	Piperine	0%	21%
HY-N0638	Dendrobine	0%	3%
HY-N1955	Protostemotinine	0%	22%
HY-N0239	Bulleyaconitine A	26%	26%
HY-N1933	Allocryptopine	11%	18%
HY-N10117	2,3-Bis(3-indolylmethyl)indole	21%	9%
HY-101407	Nicotinamide N-oxide	14%	32%
HY-N0584	Anisodamine	10%	5%
HY-N0186	Indole-3-butyric acid	2%	0%
HY-N0267	Hypaconitine	0%	6%
HY-N7649	Rhombifoline	0%	0%
HY-N0737	Harmine (hydrochloride)	22%	11%
HY-N2030	Perakine	0%	0%
HY-N0741A	Leonurine (hydrochloride)	0%	0%
HY-113412A	3-Methylhistamine (dihydrochloride)	4%	0%
HY-N0850	Benzoylhypaconine	5%	0%
HY-W002339	3-Hydroxy-2-methylpyridine	15%	0%
HY-N1431	Tabersonine	0%	1%
HY-N6825	Hydroxy-α-sanshool	3%	0%
HY-N0927	(-)-Isocorypalmine	0%	0%

HY-N2003	D-Tetrahydropalmatine	0%	0%
HY-N2517	Dihydroevocarpine	3%	0%
HY-122524	7-Methylguanosine	0%	0%
HY-N0095	(S)-10-Hydroxycamptothecin	0%	0%
HY-N4307	Laetanine	0%	0%
HY-N0654	Corypalmine	17%	16%
HY-N5121	Calycanthine	0%	0%
HY-12053A	Vinorelbine (ditartrate)	0%	10%
HY-D0143	Quinine	0%	0%
HY-I0736	Isonicotinic acid	0%	0%
HY-N9452	Capsaicin β-D-glucopyranoside	3%	0%
HY-N0770	Isoliensinine	3%	17%
HY-A0066A	Tolazoline (hydrochloride)	21%	0%
HY-125850	Berberrubine (chloride)	0%	0%
HY-N0333	Yunaconitine	24%	5%
HY-N4205	Tetrahydropiperine	0%	0%
HY-76299	Galanthamine	0%	1%
HY-N0132	Synephrine	16%	24%
HY-19332	Kifunensine	30%	5%
HY-14248	Letrozole	0%	0%
HY-120692	Cyclanoline (chloride)	7%	12%
HY-N0103	Sophocarpine	11%	9%
HY-N1103A	Vasicine (hydrochloride)	0%	2%
HY-N10127	Lepidiline C	0%	0%
HY-N2022	Castanospermine	0%	0%
HY-122267	Clovamide	2%	0%
HY-128699	D-Desthiobiotin	18%	4%
HY-13680	Meisoindigo	0%	0%
HY-N0584A	Anisodamine (hydrobromide)	0%	1%
HY-121793	Roemerine	0%	0%
HY-65008	N-Demethylricinine	0%	7%
HY-W021267	3-Demethylcolchicine	0%	0%
HY-N7596	Isoverticine	0%	0%
HY-N1373	Sophoridine	10%	0%
HY-N0214	Peimisine	0%	0%
HY-N7004	Arborine	0%	4%
HY-N0361	Dihydrocapsaicin	0%	7%
HY-B0573B	Propranolol	0%	0%
HY-121118	Coronaridine	0%	5%
HY-116474	Viridicatol	0%	0%
HY-N0775	Isocorynoxeine	0%	0%
HY-W052144	(±) Anabasine	3%	0%
HY-B0150	Nicotinamide	4%	0%
HY-N6012	(-)-Alkannin	12%	7%

HY-W040790	2,6-Dimethylpyrazine	0%	0%
HY-N6771	Cyclopiazonic acid	6%	2%
HY-121027	Anagyrine	14%	6%
HY-16560	Camptothecin	25%	6%
HY-B0573	Propranolol (hydrochloride)	0%	51%
HY-B1181	Hydrastinine (hydrochloride)	7%	0%
HY-N0696	Sipeimine	0%	9%
HY-N2037A	Higenamine (hydrochloride)	0%	7%
HY-N7203	N-Caffeoyl O-methyltyramine	0%	0%
HY-18258	Berberine (chloride)	0%	0%
HY-N6828	Monocrotaline N-Oxide	0%	0%
HY-N0674A	Dehydrocorydaline (chloride)	0%	0%
HY-N3035	Tetrahydroepiberberine	10%	3%
HY-N0049	Nuciferine	0%	16%
HY-N0170	Indole-3-carbinol	0%	18%
HY-107854	N-Acetyl-5-hydroxytryptamine	0%	0%
HY-19474	Maytansinol	0%	0%
HY-N8505	Lobelanine	0%	5%
HY-N0480A	Reserpine (hydrochloride)	0%	0%
HY-100309	9-Aminocamptothecin	0%	2%
HY-N0219	Bicuculline	0%	8%
HY-128553	Antineoplaston A10	0%	0%
HY-N1440	Koumine	16%	18%
HY-N0277	Aconine	10%	0%
HY-N0110	Palmatine (chloride)	19%	0%
HY-N4030	Humantenmine	8%	12%
HY-N0471	L-Hyoscyamine	16%	12%
HY-12882A	Ifenprodil (tartrate)	12%	15%
HY-15758	3,3'-Diindolylmethane	0%	7%
HY-N2164	3-Deoxyaconitine	9%	0%
HY-121376	Neoamygdalin	0%	0%
HY-N4238	Dehydrocorydaline (nitrate)	5%	0%
HY-N9251	Cyclo(Ile-Ala)	16%	22%
HY-18569	3-Indoleacetic acid	4%	11%
HY-119502	Camalexin	50%	27%
HY-107276	Yubeinine	13%	0%
HY-N0224	Epigoitrin	6%	0%
HY-N0114A	(±)-Evodiamine	0%	12%
HY-N1924	Crassicauline A	0%	0%
HY-N3945	Glaucine	0%	11%
HY-N0147	Rutaecarpine	0%	24%
HY-N0110B	Palmatine (hydroxide)	1%	0%
HY-12710A	Rauwolscine (hydrochloride)	7%	0%
HY-13744	Rubitecan	5%	0%

HY-W015815	6-Methylnicotinamide	0%	0%
HY-N7061	Tropine	9%	0%
HY-N2411	Geissoschizine methyl ether	0%	19%
HY-W008350	(+)-Sparteine	5%	13%
HY-17568	Nonivamide	12%	0%
HY-B0433A	Quinine (hydrochloride dihydrate)	10%	8%
HY-N6972	Cepharanthine	6%	21%
HY-W018601	Nortropine	12%	11%
HY-N0931	Santacruzamate A	0%	0%
HY-107670	Dihydro-β-erythroidine (hydrobromide)	7%	6%
HY-N0190	Amygdalin	13%	0%
HY-N2307A	Lirinidine	0%	6%
HY-107339	Deserpidine	6%	21%
HY-N2108	7-Ethylcamptothecin	11%	22%
HY-N2909	Aurantiamide	0%	15%
HY-N6778	Paxilline	12%	0%
HY-N2063	12-Ethyl-9-hydroxycamptothecin	16%	27%
HY-W011151	trans-Zeatinriboside	9%	0%
HY-N7608	Beiwutine	21%	13%
HY-W110662	Transtorine	0%	0%
HY-N0289	Lycorine (hydrochloride)	15%	0%
HY-N0212	Peimine	7%	0%
HY-N2129	N-Nornuciferine	13%	0%
HY-17577	Berberine (chloride hydrate)	0%	0%
HY-119674A	Xanthopterin (hydrate)	5%	0%
HY-B1532	Anabasine	0%	0%
HY-N5025	Bullatine A	0%	3%
HY-119529	Jineol	0%	0%
HY-N0485	Liensinine (Diperchlorate)	0%	0%
HY-12715	Yohimbine	7%	0%
HY-N0282	Colcemid	0%	0%
HY-113382	N-Methylhydantoin	13%	16%
HY-107383	Tetrahydrobiopterin	0%	3%
HY-N2368A	Arecaidine (hydrochloride)	7%	6%
HY-77817	Pyrrole-2-carboxaldehyde	0%	6%
HY-N2616	Vomicine	0%	0%
HY-76705	Methyl N-methylanthranilate	30%	4%
HY-N0226A	Epiberberine (chloride)	22%	8%
HY-N2162	12-Epinapelline	0%	9%
HY-N0387	Rhynchophylline	0%	8%
HY-N0793	Protopine	0%	0%
HY-N6011	9-Methoxycamptothecin	22%	0%
HY-B0459	Scopine	13%	5%
HY-40161	Indole-3-carboxylic acid	0%	0%

HY-N6932	Voacamine	0%	0%
HY-N2384A	Febrifugine (dihydrochloride)	30%	2%
HY-N1089	Vincosamide	6%	13%
HY-N0837	Veratramine	9%	2%
HY-N5077	Sinapine	0%	1%
HY-N2380	N-Benzoyl-(2R,3S)-3-phenylisoserine	54%	29%
HY-B1178	Cotinine	45%	16%
HY-N2080	Songorine	5%	9%
HY-N0352	Tuberostemonine	6%	4%
HY-N1599	16-Epivoacarpine	7%	2%
HY-112642	9-Methoxycanthin-6-one	0%	22%
HY-N0276	Flaconitine	13%	0%
HY-N8728	Aposcopolamine	14%	10%
HY-N4188	N-Benzyloctadecanamide	35%	17%
HY-N2368	Arecaidine	16%	0%
HY-N2060	Evocarpine	8%	20%
HY-N0687	Vindoline	14%	0%
HY-113236	p-Synephrine	13%	0%
HY-13417A	AICAR (phosphate)	20%	6%
HY-N0107	Cyclovirobuxine D	20%	0%
HY-N3196	Neotuberostemonine	0%	9%
HY-W007376	Indole-3-carboxaldehyde	0%	17%
HY-N2309	Kainic acid	0%	0%
HY-W012683	Iminodiacetic acid	15%	0%
HY-N0446	10-Methoxycamptothecin	0%	0%
HY-N7010	(-)-Corynoxidine	19%	10%
HY-N0535	(+)-Magnoflorine (chloride)	0%	0%
HY-136648A	2'-Deoxyadenosine-5'-triphosphate (trisodium)	21%	0%
HY-N0925	Tetrahydroberberine	2%	0%
HY-N2393	Kukoamine B	8%	0%
HY-W104368	Nicotinic acid riboside	5%	0%
HY-N1198	Strictosamide	0%	9%
HY-N0488	Vincristine (sulfate)	0%	0%
HY-14860	1-Deoxynojirimycin	0%	1%
HY-W098556	4-Hydroxyhygric acid	21%	0%
HY-N0404	Sinigrin	0%	0%
HY-N1914	Ergothioneine	16%	0%
HY-N5079	Lotaustralin	11%	20%
HY-N0415	Trigonelline (chloride)	2%	17%
HY-A0129	Histamine (phosphate)	6%	0%
HY-N0218	Benzoylmesaconine	1%	0%
HY-40135	L-Hydroxyproline,BioReagent, suitable for cell culture	11%	0%
HY-N0749A	Jatrorrhizine (hydroxide)	8%	5%

HY-N0924	(±)-Stylopine	0%	31%
HY-N0836	Jervine	0%	33%
HY-N0498	Nitidine (chloride)	0%	7%
HY-N0935	Ligustrazine (hydrochloride)	0%	17%
HY-N2560	Senecionine	0%	24%
HY-N6894A	Pseudocoptisine (chloride)	0%	14%
HY-N0724	Mesaconitine	0%	3%
HY-N5014	Liensinine (perchlorate)	7%	0%
HY-13764	Tetrandrine	0%	16%
HY-N0430A	Coptisine (Sulfate)	0%	9%
HY-N0592A	Demethyleneberberine (chloride)	0%	13%
HY-N1436	L-(+)-Abrine	0%	0%
HY-N0741	Leonurine	0%	0%
HY-N0052A	Sanguinarine (chloride)	0%	20%
HY-N6865	Groenlandicine	0%	0%
HY-N2005	Cycleanine	4%	4%
HY-N0590	Corynoxeine	0%	2%
HY-N0068	Solasodine	12%	20%
HY-N2079	(-)-Securinine	0%	12%
HY-N2365	N-Benzylpalmitamide	0%	20%
HY-N8346	8-Oxocoptisine	2%	30%
HY-100806	Kynurenic acid	17%	0%
HY-N0789	Delsoline	5%	0%
HY-B0739A	Citicoline (sodium)	4%	0%
HY-N2361	N-Benzyllinoleamide	0%	0%
HY-N0300A	Tetrahydropalmatine (hydrochloride)	0%	9%
HY-N0714	Berbamine	0%	0%
HY-N5022	Evolitrine	0%	15%
HY-123033A	Nicotinamide riboside (chloride)	15%	0%
HY-N0217	Benzoylaconine	2%	0%
HY-N8157	4'-O-Methylpyridoxine	26%	1%
HY-107275	Ebeiedinone	0%	4%
HY-N0682	Pyridoxine (hydrochloride)	31%	1%
HY-N0926	Columbamine	1%	0%
HY-W001542	5-Hydroxyoxindole	15%	10%
HY-B0762	Acetyl-L-carnitine (hydrochloride)	12%	0%
HY-N0300	Tetrahydropalmatine	10%	3%
HY-12048	Chelerythrine (chloride)	17%	22%
HY-N3184	N-Methylflindersine	0%	1%
HY-N0510	Aristolochic acid A	12%	22%
HY-B1021	Vincamine	0%	3%
HY-N2373A	Palmaturbine (hydroxide)	0%	8%
HY-N0127	Yohimbine (Hydrochloride)	24%	0%
HY-N0746	Oxysophocarpine	34%	0%

HY-N0221	Daurisoline	23%	12%
HY-N6827	Usaramine N-oxide	22%	14%
HY-13715A	Norepinephrine (hydrochloride)	23%	0%
HY-N0737A	Harmine	36%	4%
HY-107271	Imperialine 3-β-D-glucoside	10%	10%
HY-N1483	Guanfu base A	0%	0%
HY-17470	Mizoribine	28%	29%
HY-N6824	Hydroxy-β-sanshool	46%	10%
HY-W001909	Myosmine	18%	24%
HY-13768A	Topotecan (Hydrochloride)	5%	3%
HY-N2260	Cephaeline (dihydrochloride)	18%	4%
HY-N2377	Allosecurinine	23%	29%
HY-Y1129	3-Hydroxypyridine	18%	19%
HY-N1584	Halofuginone	18%	11%
HY-76228	1H-pyrazole	0%	21%
HY-N0114	Evodiamine	0%	23%
HY-N1637	1-Methyl-2-pentyl-4(1H)-quinolinone	44%	30%
HY-N0902	Dihydrosanguinarine	0%	0%
HY-P1940	Maculosin	0%	0%
HY-16563	Narciclasine	32%	14%
HY-N0175	Cytisinicline	29%	0%
HY-N3536	Canthin-6-one	0%	15%
HY-N2232	N-Feruloyloctopamine	14%	0%
HY-101397	Allopurinol riboside	0%	0%
HY-B0811	Salicylamide	2%	0%
HY-N7612	N-Desmethyl Galanthamine	0%	0%
HY-121936	Yohimbic acid	4%	51%
HY-N6029	Dehydroevodiamine (hydrochloride)	8%	1%
HY-Y0152	Cinchonine	6%	0%
HY-13738A	Raloxifene (hydrochloride)	32%	0%
HY-N1372A	Fangchinoline	0%	0%
HY-W007324	Maleimide	25%	32%
HY-N0736	Coptisine (chloride)	1%	6%
HY-107811	Harmol	0%	0%
HY-N0923	Corydaline	0%	8%
HY-N0759	Acetylcorynoline	34%	11%
HY-N0213	Peiminine	0%	19%
HY-N0674	Dehydrocorydaline	4%	0%
HY-N0414	Trigonelline	0%	0%
HY-N7625	N-Formylcytisine	0%	0%
HY-N3033	N-Benzyllinolenamide	2%	0%
HY-33037	Phenazine-1-carboxylic acid	0%	11%
HY-N0901	Corynoxine	3%	3%
HY-N4309A	Lotusine (hydroxide)	4%	0%

HY-14944	Homoharringtonine	20%	2%
HY-13516	Aloperine	20%	0%
HY-N0663	Talatisamine	6%	0%
HY-N0480	Reserpine	39%	16%
HY-N6637	Senecionine N-oxide	39%	43%
HY-118824A	N-Feruloylserotonin	11%	18%
HY-W009783	1-Deoxymannojirimycin (hydrochloride)	32%	11%
HY-122489	DL-Laudanosine	7%	12%
HY-N0113	Hordenine	28%	10%
HY-118341	Clitocine	0%	4%
HY-N0750	Monocrotaline	12%	12%
HY-N0484	Liensinine	34%	8%
HY-N0164	Matrine	35%	2%
HY-17439	Salinomycin (sodium salt)	28%	18%
HY-B0912	Piperazine	3%	0%
HY-10240	R-7128	33%	3%
HY-B0133	Natamycin	26%	0%
HY-B1275	Cephalothin (sodium)	9%	11%
HY-B1119	Triclosan	45%	60%
HY-D0143	Quinine	27%	8%
HY-B1805	Triclocarban	15%	20%
HY-10373	Trimetrexate	15%	0%
HY-10392	Sutezolid	16%	4%
HY-N0402	Artemether	33%	9%
HY-B0996	Hexetidine	46%	39%
HY-14904A	Arbidol (hydrochloride)	33%	3%
HY-B0467B	Amoxicillin (trihydrate)	27%	0%
HY-13238	Dolutegravir	25%	3%
HY-B1908	Midecamycin	16%	14%
HY-17561	G-418 (disulfate)	26%	11%
HY-10004	Faropenem daloxate	5%	17%
HY-B1463	Penicillin G sodium salt	16%	22%
HY-B0852	Tebuconazole	32%	1%
HY-50001	Nucleozin	17%	8%
HY-B1036	Decoquinate	13%	1%
HY-B1407	Phthalylsulfathiazole	12%	0%
HY-B0343A	Sarafloxacin (hydrochloride)	32%	3%
HY-17452A	Cefditoren (Pivoxil)	36%	1%
HY-10574	Rilpivirine	32%	20%
HY-N2301	Pleuromutilin	33%	10%
HY-13998	ABT-333	40%	0%
HY-B2148	Myclobutanil	30%	12%
HY-104032	Ac-CoA Synthase Inhibitor1	16%	8%
HY-15592	Cabotegravir	19%	7%

HY-B0724A	Pazufloxacin (mesylate)	28%	2%
HY-17423A	Abacavir (sulfate)	29%	2%
HY-B0479	Thiamphenicol	24%	1%
HY-B0307	Idoxuridine	38%	11%
HY-100593	Spiramycin	42%	80%
HY-B0529A	Azlocillin (sodium salt)	31%	0%
HY-10465	Daclatasvir (dihydrochloride)	39%	2%
HY-15303	BAY 57-1293	36%	0%
HY-14134	BMS-378806	47%	41%
HY-B1345	Piroctone olamine	5%	7%
HY-B1596A	Ceftizoxime (sodium)	0%	3%
HY-B0438	Spectinomycin (dihydrochloride)	0%	1%
HY-B0359	Fenticonazole (Nitrate)	45%	13%
HY-B0509B	Amikacin (sulfate)	0%	3%
HY-17591	Penicillin G (potassium)	14%	8%
HY-B1218	Sulfaphenazole	0%	3%
HY-15457	Triciribine	0%	0%
HY-B1282A	Sulfaquinoxaline sodium salt	87%	67%
HY-14855A	(S)-Tedizolid	26%	5%
HY-B1143	Broxaldine	26%	0%
HY-B0175	Toltrazuril	31%	2%
HY-12530	Velpatasvir	19%	1%
HY-B0136	Cefdinir	0%	0%
HY-B0914A	10-Undecenoic acid (zinc salt)	43%	35%
HY-17426	Famciclovir	15%	2%
HY-B1324	Oxiconazole nitrate	33%	16%
HY-14879A	Avibactam (sodium)	0%	8%
HY-15005	PSI-7977	6%	11%
HY-B0937A	Amprolium (hydrochloride)	12%	0%
HY-B2170A	Octenidine (dihydrochloride)	84%	96%
HY-10235	Telaprevir	7%	0%
HY-13269	BMS-707035	0%	1%
HY-100180	Delpazolid	2%	3%
HY-12320	Cycloheximide	17%	0%
HY-13234	Rifaximin	7%	1%
HY-B1864B	Kasugamycin (hydrochloride hydrate)	12%	0%
HY-B0186B	Cefoselis (sulfate)	8%	0%
HY-13557	Ascomycin	6%	0%
HY-17007	Saquinavir	37%	0%
HY-76200	Voriconazole	19%	0%
HY-102014	RN-18	20%	0%
HY-17396	Butenafine (Hydrochloride)	43%	0%
HY-B0425A	Novobiocin (Sodium)	25%	1%
HY-A0277	Cyproconazole	42%	0%

HY-B0525A	Carbenicillin (disodium)	27%	27%
HY-17437A	Mefloquine (hydrochloride)	45%	22%
HY-B0272	Rifampicin	13%	4%
HY-B0974	Methicillin (sodium salt)	16%	1%
HY-16980A	Eravacycline (dihydrochloride)	35%	27%
HY-B0174	Olsalazine (Disodium)	6%	12%
HY-B2174	Ethacridine (lactate)	66%	87%
HY-13578	Brivudine	6%	14%
HY-B0222	1-Docosanol	33%	25%
HY-B1703	Nifursol	8%	6%
HY-B0269	Rifapentine	9%	13%
HY-101506	2,4,6-Tribromophenyl caproate	48%	57%
HY-13698	Nandrolone decanoate	29%	31%
HY-B0147A	Pefloxacin (mesylate)	9%	8%
HY-B0458	Cefprozil (monohydrate)	12%	14%
HY-B0139	Flucytosine	23%	32%
HY-B1414	Chloroxylenol	100%	100%
HY-18715	Ornidazole (Levo-)	27%	23%
HY-B1620	Polyvinylpyrrolidone	10%	4%
HY-B2232	Benzalkonium chloride	50%	58%
HY-B0963	Cloxiquine	40%	19%
HY-17580	Fidaxomicin	82%	88%
HY-18982	Anisomycin	10%	44%
HY-16438	RRx-001	19%	6%
HY-17422	Acyclovir	17%	12%
HY-B0322	Sulfamethoxazole	30%	11%
HY-B0712B	Ceftriaxone (sodium salt)	25%	16%
HY-B1235	Acetohydroxamic acid	13%	4%
HY-B1040	Ftaxilide	18%	10%
HY-B0187A	Doripenem (monohydrate)	15%	7%
HY-B0226	Nitrofurazone	25%	22%
HY-I0447A	p-Aminosalicylic acid (sodium salt dihydrate)	24%	6%
HY-B0978	DEET	59%	25%
HY-B1336	Furazolidone	21%	7%
HY-10571A	Delavirdine (mesylate)	22%	16%
HY-17424	Penciclovir	22%	1%
HY-B0470	Neomycin (sulfate)	21%	3%
HY-B1381	Cefixime	38%	12%
HY-15236	PSI-6206	19%	5%
HY-B0806	Proguanil	36%	6%
HY-12643	Eprinomectin	96%	86%
HY-B0519A	Tylosin	22%	0%
HY-B0250	Lamivudine	23%	5%
HY-B0338A	Rimantadine (hydrochloride)	45%	25%

HY-B0117	Tigecycline	31%	19%
HY-B1418	Tazobactam	16%	21%
HY-A0088	Cefotaxime (sodium salt)	30%	34%
HY-B0101	Fluconazole	7%	12%
HY-13782	Tenofovir (Disoproxil Fumarate)	5%	1%
HY-17594	Oxyclozanide	10%	93%
HY-15310	Doramectin	66%	49%
HY-17003	Saquinavir (Mesylate)	0%	10%
HY-17035	Ivermectin	98%	85%
HY-17427	Emtricitabine	26%	32%
HY-10468	NM107	37%	27%
HY-B0689A	Indinavir (sulfate)	8%	10%
HY-14740	Elvitegravir	23%	48%
HY-B0555A	Nafcillin (sodium monohydrate)	12%	2%
HY-10980	AN-2690	0%	12%
HY-77036	Furagin	6%	0%
HY-B0333	Sulfamethizole	4%	0%
HY-B0916	Propoxur	16%	9%
HY-B1408	Salicylanilide	43%	21%
HY-B0273	Sulfadiazine	14%	7%
HY-13666	Levamisole (hydrochloride)	87%	61%
HY-B1267	Sulfaguanidine	22%	8%
HY-90005	Etravirine	17%	2%
HY-B0508	Ornidazole	8%	7%
HY-17514	Itraconazole	18%	22%
HY-17442	Azathramycin	11%	10%
HY-B0276	Ethionamide	64%	12%
HY-B0898	Ceftiofur (sodium)	17%	1%
HY-101476	Emodepside	69%	47%
HY-76210	5-hydroxypyrazine-2-carboxylic acid	28%	1%
HY-13678A	Meropenem (trihydrate)	12%	9%
HY-B0608	Chlorhexidine (digluconate)	39%	46%
HY-13025	HIV-1 integrase inhibitor	22%	10%
HY-B1118	Secnidazole	26%	3%
HY-B0116	Stavudine	25%	3%
HY-B0318	Metronidazole	34%	14%
HY-P1350	H-Lys-Trp-Lys-OH	55%	10%
HY-B0453	Econazole (nitrate)	35%	13%
HY-B0129	Aztreonam	14%	3%
HY-B0242	Sulfanilamide	21%	7%
HY-B0402A	Amantadine (hydrochloride)	42%	2%
HY-B0323	Sulfisoxazole	9%	7%
HY-100126	Tubercidin	5%	0%
HY-13859	Clevudine	2%	0%

HY-17413	Zidovudine	7%	4%
HY-B0827	Dinotefuran	7%	0%
HY-B0420A	Moroxydine (hydrochloride)	13%	8%
HY-B0771A	Cefozopran (hydrochloride)	10%	12%
HY-B0319	Tioconazole	30%	43%
HY-B0277	Vidarabine	4%	0%
HY-B1744	Pyridoxal phosphate	11%	4%
HY-17392	Zalcitabine	19%	20%
HY-B1004	Dinitolmide	16%	14%
HY-B0306	Prothionamide	44%	7%
HY-B0466	Cloxacillin (sodium monohydrate)	14%	4%
HY-B0220	Erythromycin	10%	5%
HY-B1256	Cefuroxime (sodium)	19%	17%
HY-B1194	Tetramisole (hydrochloride)	78%	82%
HY-B1400	Diiodohydroxyquinoline	38%	40%
HY-108365	Gamithromycin	37%	44%
HY-B0875	Cefmenoxime (hydrochloride)	15%	0%
HY-B0308	Sparfloxacin	37%	11%
HY-B0200	Cephalexin	21%	13%
HY-B0126	Marbofloxacin	23%	9%
HY-18702	Targocil	21%	11%
HY-B0467	Amoxicillin (sodium)	29%	10%
HY-B0474	Tetracycline (hydrochloride)	26%	11%
HY-B1248	Chlorhexidine	38%	63%
HY-B0454A	Miconazole (nitrate)	26%	15%
HY-13623A	Entecavir (monohydrate)	18%	3%
HY-14266	Dapivirine	44%	4%
HY-100666	Fosfluconazole	17%	0%
HY-B0398	Nalidixic acid	27%	18%
HY-B1228	Ribostamycin (sulfate)	33%	13%
HY-B1174	Bekanamycin	23%	10%
HY-B0777	Moxidectin	99%	86%
HY-B0957	Erythromycin Ethylsuccinate	24%	7%
HY-B0433A	Quinine (hydrochloride dihydrate)	38%	8%
HY-B0736A	Sertaconazole (nitrate)	40%	6%
HY-17516	Tolfenpyrad	80%	98%
HY-B1156	Cefradine	37%	11%
HY-B1075	Fosfomycin (calcium)	4%	0%
HY-B2138	Ethopabate	17%	1%
HY-B0565	Ronidazole	9%	0%
HY-15602A	Ledipasvir (acetone)	14%	4%
HY-10466	Daclatasvir	44%	43%
HY-B1300	Cefonicid (sodium)	0%	2%
HY-B0434	Ribavirin	91%	76%

HY-B0905A	Tilmicosin (phosphate)	6%	10%
HY-16349	Nimorazole	0%	0%
HY-B1340	Carbadox	4%	0%
HY-101401	3-(Methylthio)propionic acid	9%	0%
HY-B1117	Cefoxitin (sodium)	0%	0%
HY-B2116	Osalmid	48%	1%
HY-17506	Azithromycin	23%	37%
HY-B1088	Clopidol	7%	11%
HY-13750	Ebselen	13%	12%
HY-100833	Antibiotic-5d	0%	0%
HY-B1948	Diniconazole	19%	5%
HY-B0239	Chloramphenicol	6%	2%
HY-B1751	Quinidine	23%	3%
HY-19952	Pleconaril	4%	14%
HY-A0076	Tebipenem	0%	7%
HY-B1071A	Lasalocid (sodium)	15%	4%
HY-B0798	Cefpiramide (sodium)	0%	7%
HY-15662	Tulathromycin A	20%	9%
HY-B0244	Praziquantel	27%	0%
HY-17015	Peramivir (trihydrate)	2%	0%
HY-B0105	Ketoconazole	13%	0%
HY-B0268A	Enoxacin (hydrate)	17%	0%
HY-B0159	Balofloxacin	0%	20%
HY-B0357	Diclazuril	28%	44%
HY-B1370	Hydroxychloroquine sulfate	19%	9%
HY-B1828A	Spectinomycin (dihydrochloride pentahydrate)	16%	2%
HY-14603	Clioquinol	23%	0%
HY-B0200B	Cephalexin (monohydrate)	8%	0%
HY-B0455	Lomefloxacin (hydrochloride)	39%	0%
HY-B0490	Hygromycin B	67%	89%
HY-B1824	Cefpirome (sulfate)	13%	0%
HY-15602	Ledipasvir	25%	1%
HY-15287A	Nelfinavir (Mesylate)	47%	5%
HY-17423	Abacavir	3%	2%
HY-104074	Pocapavir	29%	18%
HY-B0213	Sulfameter	25%	8%
HY-B1210	Pipemidic acid	31%	14%
HY-13573	Biapenem	18%	3%
HY-A0032A	Valganciclovir (hydrochloride)	9%	0%
HY-A0278	Hexaconazole	14%	22%
HY-B0643	Dirithromycin	3%	0%
HY-13582	Carbendazim	18%	7%
HY-13210	Zanamivir	22%	6%
HY-17595	Mebendazole	18%	37%

HY-B1444	Isoconazole (nitrate)	17%	4%
HY-B0291	Oxfendazole	12%	5%
HY-B0370	Tolnaftate	25%	26%
HY-10882	Clotrimazole	6%	0%
HY-B0439	Sulfadoxine	18%	5%
HY-B1327	Chlortetracycline (hydrochloride)	24%	5%
HY-B1329	Apramycin (sulfate)	29%	2%
HY-B0210	Cefoperazone	22%	7%
HY-B0435	Roxithromycin	22%	4%
HY-B0413	Fenbendazole	8%	14%
HY-B0238	Amorolfine (hydrochloride)	20%	9%
HY-B1043	Piromidic acid	9%	8%
HY-76648	NBD-556	45%	11%
HY-B0301	Bifonazole	25%	31%
HY-B0912B	Piperazine (malate)	26%	14%
HY-B0956	Paromomycin (sulfate)	14%	2%
HY-12687	Tizoxanide	40%	53%
HY-B1902	Diaveridine	17%	32%
HY-B0465	Oxacillin (sodium monohydrate)	27%	9%
HY-B0293	Butoconazole (nitrate)	45%	46%
HY-B0614A	Mafenide (Acetate)	21%	10%
HY-B0945	Nitromide	25%	12%
HY-17025	Rifabutin	41%	2%
HY-A0090	Nitrofurantoin	24%	4%
HY-B0576	Sulfacetamide (Sodium)	33%	6%
HY-B1802A	Tosufloxacin (tosylate hydrate)	44%	36%
HY-107373	β-Chloro-L-alanine	27%	7%
HY-B0295	Chloroxine	43%	34%
HY-75800	VX-222	48%	57%
HY-17438	Cidofovir	15%	3%
HY-B1166	Cefamandole (nafate)	0%	0%
HY-13832	Atovaquone	18%	2%
HY-10581	Gatifloxacin	0%	0%
HY-B0803	Lumefantrine	0%	0%
HY-A0089	Colistin (sulfate)	14%	0%
HY-14588	Lopinavir	17%	14%
HY-12353A	Pimodivir	7%	9%
HY-B0221	Amphotericin B	2%	29%
HY-13625	Ertapenem sodium	0%	0%
HY-B0506	Nadifloxacin	0%	0%
HY-B0330	Levofloxacin	9%	10%
HY-P0017	Aprotinin	4%	9%
HY-14855	Tedizolid	20%	12%
HY-10522	HIV-1 integrase inhibitor 2	14%	13%

HY-10353A	Raltegravir (potassium salt)	3%	4%
HY-B0125	Ofloxacin	2%	0%
HY-B0449	Methacycline (hydrochloride)	17%	20%
HY-B1212	Broxyquinoline	0%	8%
HY-17583	Griseofulvin	3%	1%
HY-17518	Valifenalate	26%	3%
HY-17581	Buparvaquone	6%	12%
HY-12993A	RSV604 (racemate)	23%	13%
HY-B0299	Oxibendazole	17%	28%
HY-B0334	Sulbactam	1%	9%
HY-B0030	D-Cycloserine	12%	13%
HY-17036	Naphthoquine (phosphate)	28%	8%
HY-B0501	Danofloxacin (mesylate)	0%	15%
HY-B1790	Terconazole	26%	3%
HY-17566	Capreomycin (sulfate)	12%	0%
HY-17008	Toltrazuril (sulfone)	38%	12%
HY-B0376	Mecarbinate	38%	36%
HY-11097	TMC353121	25%	4%
HY-12640	Pyrantel (pamoate)	94%	18%
HY-76293	I2906	27%	30%
HY-P0052	Enfuvirtide	36%	30%
HY-B0955	Oxethazaine	44%	25%
HY-10846	Delamanid	42%	21%
HY-B0472	Streptomycin (sulfate)	31%	26%
HY-B1344	Oxantel (pamoate)	54%	33%
HY-B0883	Proflavine (hemisulfate)	7%	16%
HY-B0024	Prulifloxacin	27%	0%
HY-100436	Cadazolid	5%	31%
HY-B0810A	Pivmecillinam (hydrochloride)	12%	0%
HY-17028	Besifloxacin (Hydrochloride)	6%	32%
HY-B1434	7-Aminocephalosporanic acid	3%	0%
HY-B0616	Cefepime (Dihydrochloride Monohydrate)	7%	1%
HY-17016	Oseltamivir (phosphate)	0%	0%
HY-13238A	Dolutegravir (sodium)	0%	17%
HY-B0441	Tobramycin	9%	0%
HY-16566A	Kanamycin (sulfate)	22%	7%
HY-10241	Simeprevir	2%	25%
HY-B1056	Procodazole	0%	3%
HY-10529	Betulinic acid	27%	2%
HY-B1222	Sisomicin (sulfate)	2%	0%
HY-B0992	Nithiamide	32%	7%
HY-N0084	Betulinaldehyde	7%	3%
HY-15232	GS-7340	11%	1%
HY-B1781	Sulfachloropyridazine	14%	0%

HY-A0059	Nifuratel	0%	4%
HY-19285	Sulfaclozine	3%	1%
HY-B1387	Sulfamethoxypyridazine	2%	8%
HY-13318	Oseltamivir (acid)	18%	8%
HY-B1257	Cefmetazole (sodium)	7%	25%
HY-B2186	Piperazine adipate	10%	13%
HY-B2053	Tolclofos-methyl	35%	20%
HY-13910	Tenofovir	22%	16%
HY-13637	Ganciclovir	22%	5%
HY-17373	Posaconazole	23%	0%
HY-B0027	Valnemulin (Hydrochloride)	29%	12%
HY-B0396	Tebipenem pivoxil	55%	37%
HY-B1151	Climbazole	53%	2%
HY-13231	CDK9-IN-1	54%	3%
HY-B0535A	Ethambutol (dihydrochloride)	26%	6%
HY-15233	Letermovir	24%	0%
HY-B0408A	Clindamycin (hydrochloride)	28%	3%
HY-12651	Primaquine (Diphosphate)	37%	0%
HY-B0017	Telbivudine	39%	1%
HY-10238	Danoprevir	60%	24%
HY-B1479B	Emetine (dihydrochloride hydrate)	52%	7%
HY-101412	N2-Methylguanine	14%	43%
HY-B0826	Spirodiclofen	5%	0%
HY-15311	Avermectin B1	81%	41%
HY-13579	BTZ043	0%	5%
HY-90001	Ritonavir	2%	0%
HY-N1487	Oleanonic acid	15%	0%
HY-B1134	Imazalil	35%	36%
HY-B1244	Dimetridazole	0%	14%
HY-A0086	Netilmicin (sulfate)	0%	1%
HY-B0990	Thiostrepton	11%	31%
HY-17598	Rafoxanide	8%	1%
HY-76649	NBD-557	22%	14%
HY-B1460A	Sulconazole (nitrate)	37%	24%
HY-10570	Nevirapine	10%	6%
HY-17367A	Atazanavir (sulfate)	3%	4%
HY-16321	Micafungin (sodium)	3%	0%
HY-A0130	Sulfalene	2%	15%
HY-A0097	Teicoplanin	3%	9%
HY-B0946	Sulfamonomethoxine	6%	1%
HY-B1160	p-Phenylene diisothiocyanate	2%	9%
HY-17596	Closantel	4%	48%
HY-17041	Darunavir (Ethanolate)	14%	10%
HY-B0450	Ciclopirox	42%	41%

HY-B0035	Sulfamethazine	5%	3%
HY-B0507A	Sulfathiazole (sodium)	15%	6%
HY-17362	Vancomycin (hydrochloride)	15%	0%
HY-B1078	Cefazolin (sodium)	14%	0%
HY-17395A	Terbinafine	31%	0%
HY-B0958	Mupirocin	10%	11%
HY-B0223	Albendazole	17%	6%
HY-B0778	Milbemycin oxime	100%	86%
HY-B1484	Moxalactam (sodium salt)	22%	8%
HY-A0107	Tetracycline	39%	46%
HY-B1217	Bronopol	19%	24%
HY-B0912A	Piperazine (hexahydrate)	58%	12%
HY-B1286	Piperacillin (sodium)	31%	2%
HY-14273	Isavuconazole	25%	0%
HY-B1173	(+)-Camphor	31%	1%
HY-12638	Dichlorophen	15%	0%
HY-B1211	Dehydroacetic acid	45%	17%
HY-B0698A	Ceftibuten (dihydrate)	19%	5%
HY-B0526	Flumequine	20%	21%
HY-B1350A	Fusidic acid (sodium salt)	14%	35%
HY-B0688	Dapsone	42%	24%
HY-N0337	Eugenol	49%	53%
HY-B0177	Tinidazole	25%	8%
HY-B0249	Didanosine	22%	7%
HY-15602B	Ledipasvir (D-tartrate)	34%	13%
HY-B0914	10-Undecenoic acid	60%	67%
HY-13553	Anidulafungin	54%	61%
HY-12642	Diethylcarbamazine (citrate)	13%	17%
HY-17411	Limonin	23%	6%
HY-B0094	Artemisinin	32%	9%
HY-B0275	Oxytetracycline	20%	13%
HY-13801	Fexinidazole	11%	29%
HY-12993	RSV604	37%	15%
HY-103697A	Gardiquimod trifluoroacetate	33%	0%
HY-B0734A	Cefotiam (hydrochloride)	66%	37%
HY-B1071	Lasalocid	23%	21%
HY-15232B	GS-7340 (hemifumarate)	21%	0%
HY-10237	Boceprevir	28%	39%
HY-17460A	Garenoxacin (Mesylate hydrate)	61%	25%
HY-101444A	PAβN (dihydrochloride)	9%	6%
HY-B1894A	Cefetamet pivoxil (hydrochloride)	55%	17%
HY-13782A	Tenofovir (Disoproxil)	23%	7%
HY-B0770	Artemotil	18%	0%
HY-17040	Darunavir	39%	26%

HY-17010	Retapamulin	27%	9%
HY-100039	YYA-021	44%	39%
HY-B1289	Cetylpyridinium (chloride monohydrate)	25%	2%
HY-B0889	Ethacridine (lactate monohydrate)	72%	89%
HY-B1150	Clofoctol	37%	44%
HY-12641	Pyrantel (tartrate)	85%	61%
HY-B0967	Phthalylsulfacetamide	32%	2%
HY-103095	AAI101	27%	0%
HY-B1374	Florfenicol	29%	9%
HY-B0522A	Ampicillin (sodium)	24%	1%
HY-10844	PA-824	33%	0%
HY-75867	M2 ion channel blocker	38%	5%
HY-14879B	Avibactam (sodium hydrate)	37%	3%
HY-B0271	Pyrazinamide	4%	13%
HY-B2060A	Tiamulin (fumarate)	9%	9%
HY-B1159	Nitroxoline	52%	88%
HY-B0512	Sulfamerazine	12%	7%
HY-12639A	Bephenium (hydroxynaphthoate)	12%	5%
HY-B0108	Daptomycin	3%	8%
HY-B0887	Permethrin	9%	7%
HY-B0519	Tylosin (tartrate)	0%	1%
HY-B1158	Imidazolidinyl urea	28%	10%
HY-B0488	Clorsulon	12%	5%
HY-B1360	Chlorquinaldol	19%	38%
HY-B1784	Sulfisomidin	26%	10%
HY-B1177	Crotamiton	23%	24%
HY-B0766	Bicyclol	23%	4%
HY-B1743A	Puromycin (Dihydrochloride)	13%	14%
HY-B1466	Mezlocillin (sodium)	0%	4%
HY-B1358	Lincomycin (hydrochloride hydrate)	1%	0%
HY-15232A	GS-7340 (fumarate)	20%	8%
HY-B0537B	Pentamidine (isethionate)	0%	0%
HY-13588	Cefsulodin (sodium)	17%	6%
HY-B0593	Ceftazidime	37%	28%
HY-17430	Amprenavir	41%	34%
HY-B0975	Penicillin V (Potassium)	23%	24%
HY-B2157	Robenidine hydrochloride	93%	80%
HY-17560	Demeclocycline (hydrochloride)	17%	12%
HY-B2004	Thifluzamide	69%	29%
HY-17425A	Valacyclovir (hydrochloride)	38%	9%
HY-B0255	Adefovir dipivoxil	28%	14%
HY-17592	Bithionol	40%	40%
HY-10544	Tegobuvir	68%	34%
HY-B0921	Succinylsulfathiazole	31%	17%

HY-17520	Penthiopyrad	21%	18%
HY-N1937	Pristimerin	29%	23%
HY-10572	Efavirenz	29%	29%
HY-B0217	Nitazoxanide	51%	49%
HY-30234A	Clemizole (hydrochloride)	25%	69%
HY-14283	Luliconazole	3%	7%
HY-B0337	Sulfadimethoxine	15%	3%
HY-14267	Lersivirine	18%	8%
HY-B1099	Hycanthone	31%	26%
HY-17006	Caspofungin (Acetate)	27%	4%
HY-14855B	Tedizolid (phosphate)	10%	12%
HY-B1241	Dihydrostreptomycin (sulfate)	0%	0%
HY-B0329	Isoniazid	26%	27%
HY-17508	Clarithromycin	12%	10%
HY-B1046	Clofazimine	6%	7%
HY-B1831A	Oritavancin (diphosphate)	34%	8%
HY-15597	Salinomycin	81%	93%
HY-18062	Pyrimethamine	35%	2%
HY-B0128	Diphylline	66%	31%
HY-13738	Raloxifene	24%	16%
HY-N0700	alpha-Asarone	49%	29%
HY-N0523	Gallic acid	66%	34%
HY-N1428A	Citric acid (monohydrate)	63%	32%
HY-100562	Flopropione	40%	18%
HY-B2123	Lactose	54%	10%
HY-B0216	Ethinylestradiol	33%	15%
HY-N6810	Thymol	54%	28%
HY-B0075	Melatonin	66%	24%
HY-N2064	Racanisodamine	69%	6%
HY-N0300A	Tetrahydropalmatine (hydrochloride)	0%	7%
HY-N1446	Oleic acid	0%	0%
HY-B1899A	Taurodeoxycholic acid (sodium hydrate)	8%	3%
HY-B0264	Guaifenesin	46%	27%
HY-N0060	Ferulic acid	58%	8%
HY-Y1683	DL-Menthol	27%	0%
HY-B0935	Benzyl benzoate	35%	36%
HY-N0018	Daidzin	47%	27%
HY-B0167	Salicylic acid	12%	8%
HY-N0683	α-Vitamin E	53%	11%
HY-B0315	Vitamin B12	34%	0%
HY-B0394	Atropine (sulfate monohydrate)	48%	4%
HY-N0471	L-Hyoscyamine	55%	28%
HY-16561	Resveratrol	82%	7%
HY-N0178	Diosmin	36%	10%

HY-B0809	Theophylline	46%	1%
HY-N0092	Inosine	34%	10%
HY-N0110	Palmatine (chloride)	65%	19%
HY-N7122	Thymopentin	36%	3%
HY-N0117	Indirubin	52%	6%
HY-N0375	18α-Glycyrrhetinic acid	39%	18%
HY-B0234	Estrone	32%	0%
HY-76299	Galanthamine	53%	7%
HY-13768A	Topotecan (hydrochloride)	19%	6%
HY-N0681	D-Pantothenic acid (hemicalcium salt)	48%	1%
HY-B0573B	Propranolol	84%	35%
HY-N1373	Sophoridine	64%	11%
HY-B0573	Propranolol (hydrochloride)	84%	35%
HY-B1337	Choline (chloride)	51%	17%
HY-N0537	Xylose	17%	11%
HY-107339	Deserpidine	53%	23%
HY-17556	Folinic acid	11%	20%
HY-10448A	Capsaicinoid	10%	30%
HY-13748	Silybin A	22%	20%
HY-B0222	1-Docosanol	7%	8%
HY-N0550	β-Pinene	2%	12%
HY-N0164	Matrine	3%	2%
HY-N0480A	Reserpine (hydrochloride)	22%	26%
HY-N0593A	Deoxycholic acid sodium salt	0%	26%
HY-13780	Vinblastine (sulfate)	8%	1%
HY-N0158	Oxymatrine	0%	6%
HY-B1279A	Metamizole (sodium)	17%	2%
HY-N0191	Andrographolide	22%	17%
HY-N0172	Caffeic acid	20%	13%
HY-B1465	1-Hexadecanol	17%	0%
HY-B1328	Pyridoxine	8%	0%
HY-B1205	Atropine	0%	8%
HY-B0469	Medroxyprogesterone acetate	11%	6%
HY-B2136	Tannic acid	3%	15%
HY-N0480	Reserpine	14%	0%
HY-110189	Pregnenolone monosulfate (sodium)	0%	0%
HY-A0009	Galanthamine (hydrobromide)	0%	0%
HY-B0151	Pregnenolone	11%	4%
HY-B1529A	Citric acid (triammonium)	8%	0%
HY-N0175	Cytisinicline	14%	3%
HY-N6972	Cepharanthine	38%	0%
HY-N0482	Phillyrin	0%	0%
HY-N0603	20(S)-Ginsenoside Rg3	0%	0%
HY-B1302	Quinidine hydrochloride monohydrate	40%	6%

HY-101036	Choline (bitartrate)	0%	0%
HY-N0623	L-Tryptophan	26%	3%
HY-B1167	Ajmaline	13%	1%
HY-B0166	L-Ascorbic acid	5%	9%
HY-75161	(-)-Menthol	7%	13%
HY-B0802A	Terbutaline	9%	2%
HY-B0997	Hydroquinidine	50%	3%
HY-B1899	Taurodeoxycholic acid	0%	7%
HY-B0722	Histamine (dihydrochloride)	0%	3%
HY-B0398	Nalidixic acid	22%	26%
HY-18258	Berberine (chloride)	25%	32%
HY-13715A	Norepinephrine (hydrochloride)	12%	15%
HY-N0017	Bergenin	21%	10%
HY-N0677	Dehydroandrographolide succinate	7%	16%
HY-N0148A	Rutin (hydrate)	2%	8%
HY-14944	Homoharringtonine	12%	0%
HY-N0717	L-Valine	0%	12%
HY-W015967	Glycolic acid	5%	6%
HY-B1063	Terpin (hydrate)	9%	5%
HY-N0714A	Berbamine (dihydrochloride)	53%	15%
HY-N1404	Sodium aescinate	0%	1%
HY-15142	Doxorubicin (hydrochloride)	13%	4%
HY-B0398A	Nalidixic acid (sodium salt)	0%	0%
HY-30151	Methoxsalen	23%	5%
HY-N0283	Diacerein	0%	8%
HY-N0343	Helicid	11%	4%
HY-N0862	Harringtonine	8%	0%
HY-N0145	Puerarin	10%	2%
HY-15122A	Sinomenine hydrochloride	17%	8%
HY-B0158	Cytidine	12%	5%
HY-B0934	Ethylparaben	32%	8%
HY-W013699	Chlorhexidine (diacetate)	38%	62%
HY-B1355A	Oxyphenbutazone	0%	0%
HY-W020044	DL-alpha-Tocopherol	5%	9%
HY-N0697	Crocin	1%	0%
HY-B1180	Vinburnine	0%	0%
HY-B1125	Glucosamine	8%	4%
HY-Y0319G	Magnesium acetate tetrahydrate	2%	0%
HY-B0094	Artemisinin	1%	0%
HY-B2201	Citric acid (trisodium)	8%	2%
HY-N0492	α-Lipoic Acid	9%	3%
HY-77591	Cysteamine (hydrochloride)	23%	6%
HY-N0709	Coumarin	12%	8%
HY-N6804	Diammonium Glycyrrhizinate	12%	4%

HY-W015410	Disodium succinate	19%	1%
HY-109500	Masoprocol	7%	17%
HY-B1761	Santonin	2%	6%
HY-N0524	Propyl gallate	1%	0%
HY-N0098	Vanillin	24%	11%
HY-N0716B	Berberine (sulfate)	15%	12%
HY-16637	Folic acid	23%	17%
HY-Y0271	Urea	10%	13%
HY-Y0479	L-Lactic acid	16%	21%
HY-N0420	Succinic acid	14%	15%
HY-N0538	Xylitol	22%	13%
HY-B0892	Benzyl alcohol	2%	4%
HY-Y0366	Lauric acid	58%	61%
HY-N1214	Squalene	4%	11%
HY-W010388	Creatine	4%	9%
HY-107324	β-Elemene	9%	9%
HY-B0660	Eicosapentaenoic Acid	0%	4%
HY-Y0252	L-Proline	0%	0%
HY-B0152A	Adenine (hydrochloride)	0%	0%
HY-B0011	Docetaxel	1%	15%
HY-N1369	Menthol	3%	17%
HY-I0960	Uracil	0%	0%
HY-B2219	Stearic acid	0%	1%
HY-N0324A	Cholic acid (sodium)	0%	5%
HY-N6951	Guaiazulene	1%	24%
HY-A0154	Deslanoside	5%	5%
HY-N0523A	Gallic acid (hydrate)	6%	7%
HY-10448	Capsaicin	0%	0%
HY-N0216	Benzoic acid	3%	6%
HY-B0430	D-Pantothenic acid	9%	10%
HY-13295	Vinpocetine	0%	4%
HY-B0914	10-Undecenoic acid	0%	6%
HY-B0152B	Adenine (hemisulfate)	3%	5%
HY-B0152	Adenine	0%	3%
HY-N0197	Baicalin	0%	15%
HY-N0680	Thiamine (hydrochloride)	2%	13%
HY-N7122A	Thymopentin (acetate)	4%	4%
HY-B0456	Riboflavin	3%	6%
HY-B1278	D-α-Tocopherol acetate	7%	22%
HY-Y0337	L-Cysteine	4%	7%
HY-W015883	Fumaric acid	8%	5%
HY-Y1311	Malic acid	2%	14%
HY-N0192	Arbutin	0%	1%
HY-N0714	Berbamine	11%	11%

HY-N0198	Nordihydroguaiaretic acid	8%	21%
HY-N0115	Gastrodin	4%	8%
HY-B0770	Artemotil	7%	2%
HY-B1157	Trioxsalen	6%	7%
HY-N0163	Magnolol	0%	14%
HY-B2223	Thiamine nitrate	12%	1%
HY-N0276	Flaconitine	5%	11%
HY-N0395	Fructose	0%	3%
HY-B1411	i-Inositol	0%	0%
HY-N0180	18β-Glycyrrhetinic acid	15%	3%
HY-B1671	(+)-Kavain	5%	13%
HY-A0181	Adenosine monophosphate	8%	6%
HY-N0187	4-Methylumbelliferone	4%	3%
HY-N0148	Rutin	4%	0%
HY-N0184	Glycyrrhizic acid	0%	0%
HY-Z0478	(-)-Limonene	0%	1%
HY-N0264	Ligustrazine	0%	2%
HY-N0124	Dioscin	12%	79%
HY-D0143	Quinine	1%	1%
HY-N0196	Baicalein	10%	12%
HY-N0471A	L-Hyoscyamine (sulfate)	1%	6%
HY-N0156	Oleanolic Acid	4%	1%
HY-N0597	Panaxatriol	1%	15%
HY-B2114	Escin	0%	8%
HY-N0682	Pyridoxine (hydrochloride)	0%	19%
HY-B0167A	Sodium Salicylate	2%	4%
HY-N0019	Daidzein	6%	14%
HY-108013	Armillarisin A	0%	4%
HY-N0060A	Ferulic acid (sodium)	10%	0%
HY-B0277	Vidarabine	0%	0%
HY-B1505	Acefylline	0%	1%
HY-B0704	Azelaic acid	0%	0%
HY-13407	Gossypol	0%	26%
HY-B1449	Uridine	0%	10%
HY-15552	Podofilox	0%	9%
HY-W013075	Rutin (trihydrate)	2%	9%
HY-N0676	Dehydroandrographolide	0%	7%
HY-76847	Chenodeoxycholic Acid	0%	5%
HY-N0139	Troxerutin	0%	0%
HY-B1266	Physostigmine (salicylate)	0%	0%
HY-N2591	Isocorydine	0%	7%
HY-N0281	Daphnetin	0%	1%
HY-N1380	Guaiacol	6%	10%
HY-N0865	Ingenol	0%	19%

HY-12715	Yohimbine	8%	18%
HY-B1357	Digitoxin	0%	3%
HY-B0923	Danthron	21%	9%
HY-N0783	Eupatilin	0%	9%
HY-16560	Camptothecin	6%	3%
HY-B1695	Methyl nicotinate	0%	0%
HY-N0176	Dihydroartemisinin	1%	2%
HY-15337	Hesperidin	11%	2%
HY-N0898	Catechin	0%	20%
HY-B0143	Niacin	0%	14%
HY-B0542	Ouabain (Octahydrate)	0%	2%
HY-N0337	Eugenol	1%	9%
HY-B0430A	D-Pantothenic acid (sodium)	1%	4%
HY-N6618	DL-Mannitol	0%	15%
HY-B0015	Paclitaxel	18%	12%
HY-30270	Mequinol	16%	11%
HY-N0437	Progesterone	0%	12%
HY-N0750	Monocrotaline	2%	15%
HY-N0510	Aristolochic acid A	0%	11%
HY-17510	Gossypol (acetic acid)	0%	7%
HY-B1030	Lanatoside C	0%	2%
HY-N0402	Artemether	15%	26%
HY-B0150	Nicotinamide	17%	10%
HY-103252	Monomethyl fumarate	0%	15%
HY-17577	Berberine (chloride hydrate)	31%	15%
HY-B0400	D-Sorbitol	0%	7%
HY-B1173	(+)-Camphor	2%	5%
HY-32735	Triptolide	15%	71%
HY-N0193	Artesunate	12%	30%
HY-B0141	Estradiol	1%	2%
HY-N0324	Cholic acid	0%	8%
HY-B1751	Quinidine (15% dihydroquinidine)	14%	13%
HY-N0053	Psoralen	14%	14%
HY-12053A	Vinorelbine (ditartrate)	0%	2%
HY-B1021	Vincamine	0%	10%
HY-N0096	Rotundine	0%	12%
HY-N0684	Vitamin K1	6%	13%
HY-B0511	Biotin	14%	12%
HY-B0412	Estriol	2%	10%
HY-17387	(-)-Huperzine A	0%	7%
HY-14649	Retinoic acid	18%	40%
HY-N0832	L-Histidine	23%	22%
HY-N0455A	L-Arginine (hydrochloride)	20%	17%
HY-B0220	Erythromycin	0%	5%

HY-17568	Nonivamide	0%	0%
HY-15459	Cabazitaxel	3%	9%
HY-N0454	DL-Arginine	4%	0%
HY-B1610	Sodium citrate (dihydrate)	32%	2%
HY-B0647	Butylphthalide	0%	18%
HY-N0300	Tetrahydropalmatine	9%	0%
HY-B0228	Adenosine	0%	9%
HY-B0399	L-Carnitine	7%	1%
HY-N0455B	L-Arginine (L-glutamate)	22%	0%
HY-16569	Colchicine	0%	6%
HY-N1428	Citric acid	0%	18%
HY-N0169	Hyodeoxycholic acid	2%	18%
HY-N0667	L-Asparagine	27%	25%
HY-Y0966	Glycine	32%	13%
HY-B0332	Menadione	0%	0%
HY-13629	Etoposide	0%	4%
HY-B1739	Pregnenolone monosulfate	0%	4%
HY-W009156	Citric acid (tripotassium hydrate)	28%	4%
HY-N0650	L-Serine	0%	0%
HY-N0488	Vincristine (sulfate)	0%	7%
HY-N0543	Allantoin	0%	13%
HY-B0425A	Novobiocin (sodium)	6%	6%
HY-N0486	L-Leucine	4%	11%
HY-B1352	L-Ornithine	2%	4%
HY-15122	Sinomenine	0%	0%
HY-N0808	Camphor	0%	5%
HY-N0159	Paeonol	0%	0%
HY-N0378	D-Mannitol	11%	0%
HY-N7092	D-Fructose	0%	15%
HY-N0666	L-Aspartic acid	20%	30%
HY-N0470	L-Lysine hydrochloride	19%	0%
HY-A0100	Thiamine monochloride	13%	6%
HY-A0181A	Adenosine 5'-monophosphate (monohydrate)	20%	2%
HY-N0469	L-Lysine	19%	2%
HY-N0122	5-Hydroxytryptophan	28%	12%
HY-Y0189	Methyl Salicylate	21%	12%
HY-B1092A	Gluconate (sodium)	15%	3%
HY-N0215	L-Phenylalanine	23%	21%
HY-N0184A	Dipotassium glycyrrhizinate	17%	3%
HY-N0067	γ-Aminobutyric acid	13%	21%
HY-N0593	Deoxycholic acid	29%	12%
HY-B0351	Taurine	8%	0%
HY-B1295	Lithium citrate (tetrahydrate)	13%	0%
HY-B0166A	L-Ascorbic acid (sodium salt)	6%	3%

HY-N0325	DL-Methionine	6%	2%
HY-N0322	Cholesterol	24%	17%
HY-14608A	L-Glutamic acid (monosodium salt)	1%	0%
HY-N0455	L-Arginine	0%	0%
HY-112790	Adenosylcobalamin	20%	22%
HY-A0129	Histamine (phosphate)	9%	12%
HY-N0390	L-Glutamine	3%	0%
HY-N0326	L-Methionine	0%	0%
HY-14608	L-Glutamic acid	3%	1%
HY-W015370	L-Lysine hydrate	8%	2%
HY-B1204	Histamine	5%	6%
HY-B1070	DL-Xylose	0%	5%
HY-N1446B	Sodium oleate	0%	0%
HY-N0771	L-Isoleucine	14%	17%
HY-B1247	Protoporphyrin IX	3%	17%
HY-B2235	Lecithin	20%	7%
HY-B2134	Casanthranol	9%	18%
HY-N0355	(+)-Catechin hydrate	12%	21%
HY-N0052A	Sanguinarine (chloride)	14%	27%
HY-B2172	Vitamin K	0%	15%
HY-B0356	Ciprofloxacin	33%	2%
HY-N0935	Ligustrazine (hydrochloride)	0%	17%
HY-N2079	(-)-Securinine	0%	5%
HY-N7073	Silymarin	0%	15%
HY-13764	Tetrandrine	7%	12%
HY-N7075	Inulin	4%	17%
HY-N0473	L-Tyrosine	34%	8%
HY-B0633A	Hyaluronic acid	0%	4%
HY-N1181	Tamarixetin	20%	10%
HY-N0628	Kaempferitrin	4%	16%
HY-N1413	Noricaritin	42%	5%
HY-N0530	Dryocrassin ABBA	12%	18%
HY-N0516	Casticin	9%	0%
HY-N0772	Isomangiferin	28%	46%
HY-N0567	Hydroxysafflor yellow A	36%	12%
HY-N0649	Narcissin	42%	25%
HY-N0713	Diosmetin-7-O-β-D-glucopyranoside	53%	10%
HY-N0804	Narirutin	39%	19%
HY-N0622	Morusin	33%	14%
HY-N0768	Isoquercitrin	31%	2%
HY-N0577	Apiin	24%	0%
HY-N0452	Hyperoside	14%	11%
HY-N0773	Isovitexin	11%	6%
HY-N0560	Oroxylin A	39%	12%

HY-N0647	Silychristin	33%	2%
HY-N1377	Nevadensin	39%	0%
HY-N0509	Astilbin	31%	0%
HY-N0636	Eriocitrin	54%	16%
HY-N1475	Nicotiflorin	4%	40%
HY-N1458	Isoschaftoside	9%	0%
HY-N0873	Sagittatoside A	43%	12%
HY-N1435	Oroxin B	0%	0%
HY-N0662	Amentoflavone	0%	0%
HY-N1454	Apigenin-7-glucuronide	23%	3%
HY-N0621	Morin	44%	17%
HY-N0752	Scutellarein	49%	3%
HY-N1457	Chrysosplenetin	17%	6%
HY-N0731	Genkwanin	34%	4%
HY-N0640	Kuromanin (chloride)	34%	5%
HY-N0460	1-Caffeoylquinic acid	24%	0%
HY-N0762	Isobavachin	32%	0%
HY-N0651	Spinosin	2%	6%
HY-N0646	Silydianin	17%	4%
HY-N0765	Isoliquiritin	24%	2%
HY-N1127	Tricin	31%	2%
HY-N1347	Robinetin	39%	7%
HY-N0526	2"-O-Galloylhyperin	32%	1%
HY-N1346	Robinin	31%	8%
HY-N0522	(-)-Gallocatechin gallate	60%	5%
HY-N0897	Corylifol A	40%	4%
HY-N0575	Pinocembrin	44%	5%
HY-N1098	Velutin	11%	19%
HY-N0898A	(-)-Catechin	8%	9%
HY-N1067	Xanthohumol	23%	13%
HY-N0521	(-)-Gallocatechin	43%	5%
HY-N0941	beta-Mangostin	30%	0%
HY-N1318	Salvigenin	22%	3%
HY-N0778	Isorhamnetin-3-O-neohespeidoside	45%	5%
HY-N0259	Epimedin B	15%	32%
HY-N0400	Wogonin	23%	5%
HY-N0875	IKarisoside A	32%	5%
HY-N0767	Isoorientin	6%	22%
HY-N1549	Prunin	13%	2%
HY-N0889	Ginkgetin	13%	16%
HY-N0751	Scutellarin	31%	1%
HY-N1231	Sophoraflavanone G	55%	4%
HY-N0520	Calycosin-7-O-β-D-glucoside	20%	11%
HY-15449	Kaempferide	49%	0%

HY-N0018	Daidzin	26%	12%
HY-B1671	(+)-Kavain	38%	14%
HY-N0148	Rutin	33%	10%
HY-N0268	Irisflorentin	26%	34%
HY-N0257	Epimedin A	13%	13%
HY-N0014	Icariin	30%	12%
HY-N0145	Puerarin	38%	9%
HY-N0376	Liquiritin	45%	14%
HY-129997	Luteolinidin (chloride)	29%	8%
HY-N0019	Daidzein	46%	10%
HY-N0236	Corylin	25%	15%
HY-N0112	Dihydromyricetin	33%	16%
HY-N0012	Glycitin	30%	4%
HY-N0428	Obacunone	16%	11%
HY-107207	Kaempferol 3-neohesperidoside	17%	10%
HY-N0197	Baicalin	24%	24%
HY-N0377	Liquiritigenin	44%	28%
HY-14616	Shogaol	0%	5%
HY-N0231	Bavachalcone	39%	18%
HY-N0393	Glabridin	26%	6%
HY-N1950	Hispidulin	0%	33%
HY-N2279	Kurarinone	0%	0%
HY-N2208	4-Hydroxylonchocarpin	11%	9%
HY-N1992	Theaflavin 3,3'-digallate	0%	2%
HY-N1980	3'-Hydroxypuerarin	1%	0%
HY-N2289	2''-O-Rhamnosylicariside II	0%	13%
HY-N4182	Licochalcone E	5%	0%
HY-N6250	Isomucronulatol 7-O-glucoside	8%	10%
HY-N2597	Prunetin	0%	7%
HY-N2165	Vicenin 2	0%	3%
HY-N2481	Oroxylin A-7-O-glucuronide	0%	1%
HY-N2131	Isosakuranetin	8%	0%
HY-N3213	Naringenin triacetate	0%	14%
HY-N4314	Scutellarein tetramethyl ether	0%	0%
HY-N4136	Lonicerin	2%	2%
HY-N2224	Guaijaverin	0%	25%
HY-N2443	Tribuloside	13%	16%
HY-N2441	Methylophiopogonone A	0%	0%
HY-N2584	(2S)-Isoxanthohumol	0%	2%
HY-N4072	6''-O-Acetylglycitin	4%	5%
HY-N1968	Quercetin-3-O-β-D-glucose-7-O-β-D-gentiobiosi-den	0%	0%
HY-N2376	Chrysin-7-O-glucuronide	0%	37%
HY-N2144	7,4'-Di-O-methylapigenin	0%	9%

HY-N4247	Kuwanon G	0%	75%
HY-N2183	Baimaside	13%	0%
HY-N1939	Icariside I	0%	23%
HY-N1978	3'-Methoxypuerarin	11%	51%
HY-N5024	Gambogenic acid	1%	8%
HY-N4122	Neodiosmin	14%	6%
HY-N1910	4'-O-Methylbavachalcone	19%	3%
HY-N4296	Isosakuranin	1%	2%
HY-N4184	Licoflavone B	15%	0%
HY-N1941	Isosinensetin	0%	2%
HY-N2120	Dichotomitin	34%	22%
HY-N6596	7-Hydroxy-4H-chromen-4-one	0%	8%
HY-N3017	Artemitin	0%	20%
HY-N2562	Norwogonin	0%	12%
HY-N2160	6‴-Feruloylspinosin	9%	13%
HY-N1942	5-O-Demethylnobiletin	26%	29%
HY-N6624	Skullcapflavone II	4%	6%
HY-N0898	Catechin	21%	7%
HY-N0792	Tectorigenin	0%	0%
HY-N2343	Procyanidin A2	6%	3%
HY-N0678	Icaritin	0%	2%
HY-N0533	4”-O-Glucosylvitexin	0%	0%
HY-N0534	Vitexin-2"-O-rhamnoside	2%	52%
HY-N0625A	Alpinetin	1%	14%
HY-N1444	Complanatuside	0%	22%
HY-N0595	Genistin	29%	19%
HY-N0723	Neomangiferin	7%	17%
HY-N1425	Tiliroside	2%	9%
HY-N0703	Schaftoside	0%	3%
HY-N0521A	(+)-Gallocatechin	0%	12%
HY-N0493	Pectolinarigenin	4%	0%
HY-N0637	Eriodictyol	0%	14%
HY-N1353	Rhamnocitrin	6%	23%
HY-N0528	Linarin	15%	23%
HY-N0783	Eupatilin	6%	4%
HY-N1860	3-O-Methylquercetin	3%	16%
HY-N1201	Apigenin	12%	3%
HY-N0451	Acacetin	9%	2%
HY-N0776	Isorhamnetin	22%	2%
HY-N1510	Kaempferol 3-O-gentiobioside	0%	12%
HY-N0831	Jaceosidin	0%	5%
HY-N0546	Ligustroflavone	10%	8%
HY-N0720	Neobavaisoflavone	10%	21%
HY-N1438	Hydroxygenkwanin	11%	13%

HY-N1378	(E)-Cardamonin	13%	8%
HY-N0796	Procyanidin B2	14%	27%
HY-N0540	Cynaroside	20%	0%
HY-N1419	Vaccarin	0%	6%
HY-N0791	Tectoridin	1%	11%
HY-N1354	Reynoutrin	0%	7%
HY-N1390	Syringaldehyde	4%	3%
HY-N0712	Typhaneoside	26%	15%
HY-N1463	Luteolin 7-O-glucuronide	22%	45%
HY-N0578	Apigenin 7-glucoside	10%	40%
HY-N1504	Loureirin B	10%	0%
HY-N1505	Loureirin A	11%	0%
HY-N0519	Calycosin	16%	1%
HY-N7632	5-Desmethylsinensetin	2%	11%
HY-N7030	5,7,3',4'-Tetramethoxyflavone	0%	0%
HY-N6673	Okanin	9%	31%
HY-N7269	Luteolin 7-diglucuronide	20%	7%
HY-W006492	5a-Pregnane-3,20-dione	2%	17%
HY-N6827	Usaramine N-oxide	12%	0%
HY-Y0598	trans-Chalcone	17%	96%
HY-N7368	Hibifolin	6%	1%
HY-N7036	Rhamnetin	29%	12%
HY-N7058	cis-Jasmone	10%	9%
HY-N7108	7-Hydroxyflavone	1%	0%
HY-Y1426	2'-Hydroxyacetophenone	0%	1%
HY-N5106	(E)-Flavokawain A	33%	11%
HY-N7199	(Rac)-Hydnocarpin	7%	0%
HY-N7012	7,3',4'-Tri-O-methylluteolin	13%	21%
HY-N6629	3'-Methoxyflavonol	9%	0%
HY-N2424	Flavone	0%	5%
HY-N7176	Kaempferol 3-O-β-D-glucuronide	5%	0%
HY-N7056	4'-Hydroxychalcone	39%	15%
HY-N6896	Isoviolanthin	21%	2%
HY-N5083	Saponarin	1%	1%
HY-N4187	Licochalcone D	15%	0%
HY-N7110	6-Hydroxyflavone	14%	2%
HY-N6818	5,7,4'-Trimethoxyflavone	0%	1%
HY-N7690	3,5,7,3’,4’-Pentamethoxyflavone	2%	24%
HY-W011641	(±)-Naringenin	0%	18%
HY-N6879	DiosMetin 7-O-β-D-Glucuronide	4%	21%
HY-N6895	Violanthin	0%	19%
HY-Y0678	1,3,5-Trimethoxybenzene	1%	25%
HY-N4258	Panasenoside	6%	31%
HY-N4099	Luteolin-3-O-beta-D-glucuronide	0%	26%

HY-N2286	Kushenol I	0%	8%
HY-N2123	Neoliquiritin	0%	20%
HY-N2145	4',7-Dimethoxyisoflavone	21%	27%
HY-N2342	Procyanidin C1	2%	29%
HY-N2913	Ayanin	0%	22%
HY-N4192	Toringin	0%	2%
HY-N2600	Kuwanon H	11%	23%
HY-N2374	Eupatorin	7%	26%
HY-N2025	Oroxin A	0%	1%
HY-N4172	Tectorigenin 7-O-Xylosyl Glucoside	0%	1%
HY-N6616	Pinocembrin-7-O-β-D-glucopyranoside	0%	17%
HY-N2119	Sciadopitysin	0%	21%
HY-N1940	β-Anhydroicaritin	5%	16%
HY-N2572	Nepetin	4%	17%
HY-N1970	5,7-Dihydroxychromone	0%	0%
HY-N3460	Isorhoifolin	0%	20%
HY-N4127	3'-Demethylnobiletin	0%	0%
HY-N2068	Didymin	1%	6%
HY-N3389	Licoisoflavone A	0%	10%
HY-N1949	Homoplantaginin	19%	5%
HY-N1993	5-Methyl-7-methoxyisoflavone	16%	0%
HY-N2127	Pinostrobin	1%	7%
HY-N2192	Swertianolin	11%	0%
HY-N2117	Isoginkgetin	17%	19%
HY-N4193	Glabrol	11%	9%
HY-N2437	Methylophiopogonanone A	21%	0%
HY-N2473	Methylnissolin-3-O-glucoside	8%	7%
HY-N5116	Neoisoastilbin	0%	0%
HY-N2412	Irisolidone	0%	0%
HY-N2497	Isoliquiritin apioside	47%	8%
HY-N4089	Quercetin 3-gentiobioside	42%	0%
HY-N3513	Mulberrin	32%	5%
HY-N4310	Taxifolin 7-O-rhamnoside	33%	12%
HY-N6037	Gardenin B	6%	0%
HY-N2358	Blumeatin	21%	11%
HY-N4149	Quercetagetin	15%	10%
HY-N4167	3-O-Methylgalangin	0%	4%
HY-N2587	Irigenin	14%	3%
HY-N2897	Dihydrokaempferol	2%	0%
HY-N4090	Vicenin 3	15%	3%
HY-N1881	4',5-Dihydroxyflavone	39%	6%
HY-N2344	Procyanidin A1	23%	12%
HY-N5011	5,7-Dimethoxyflavone	37%	15%
HY-N6020B	Butin	46%	11%

HY-N2445	Flavokawain C	18%	20%
HY-N2555	Tilianin	13%	3%
HY-N4126	6-Demethoxytangeretin	31%	8%
HY-N3006	Sakuranetin	19%	18%
HY-N2038	3,5,6,7,8,3',4'-Heptemthoxyflavone	34%	6%
HY-N0382	Galangin	10%	8%
HY-N0133	Tangeretin	14%	8%
HY-18085	Quercetin	24%	11%
HY-125130	Hesperetin 7-O-glucoside	16%	14%
HY-N0222	Avicularin	33%	21%
HY-N0001	(-)-Epicatechin	1%	23%
HY-N0125	Diosmetin	13%	0%
HY-N0119	Naringin Dihydrochalcone	14%	1%
HY-N0182	Fisetin	0%	2%
HY-N0013	Vitexin	39%	0%
HY-N0419	Quercimeritrin	0%	3%
HY-N0233	Bavachin	27%	24%
HY-15337	Hesperidin	0%	0%
HY-13425	Deguelin	0%	0%
HY-N0136	Taxifolin	10%	14%
HY-N0418	Quercitrin	4%	0%
HY-N0225	(-)-Epigallocatechin	3%	0%
HY-N0234	Bavachinin	0%	2%
HY-N0406	2"-O-beta-L-galactopyranosylorientin	7%	0%
HY-N0243	Theaflavin	11%	5%
HY-N0143	Phlorizin	0%	2%
HY-107569	Garcinol	8%	3%
HY-N0168A	(Rac)-Hesperetin	0%	0%
HY-14592	Tectochrysin	0%	11%
HY-N0344	Farrerol	6%	9%
HY-N0183	Formononetin	6%	31%
HY-N0436	Engeletin	11%	9%
HY-121054	Chalcone	30%	86%
HY-N0155	Nobiletin	0%	0%
HY-N0356	(-)-Catechin gallate	3%	0%
HY-113556	Sappanone A	0%	0%
HY-13748	Silibinin	0%	0%
HY-N0297	Sinensetin	0%	0%
HY-N0146	Quercetin (dihydrate)	0%	3%
HY-N0196	Baicalein	14%	19%
HY-N0279	Cardamonin	0%	13%
HY-N0258	Epimedin A1	6%	5%
HY-128400	4'-Methoxychalcone	0%	5%
HY-N0016	Glycitein	0%	0%

HY-N0245	Theaflavin-3-gallate	8%	22%
HY-N0011	Baohuoside I	0%	0%
HY-107198	(2S)-6-Prenylnaringenin	0%	0%
HY-111806	3,7,4'-Trihydroxyflavone	0%	16%
HY-N0423	Sophoricoside	0%	9%
HY-15097	Myricetin	30%	7%
HY-130237	Cinnamtannin B-1	0%	4%
HY-N0162	Luteolin	4%	0%
HY-N0244	Theaflavin-3'-gallate	9%	0%
HY-N0269	Echinatin	17%	10%
HY-14595	Biochanin A	7%	0%
HY-N0153	Naringin	0%	3%
HY-N0374	Licochalcone C	0%	8%
HY-107818	4-Hydroxychalcone	0%	1%
HY-13653	(-)-Epigallocatechin Gallate	9%	6%
HY-14596	Genistein	5%	2%
HY-121471	Chrysoeriol	18%	1%
HY-N0260	Epmedin C	0%	0%
HY-107825	Flavonol	2%	10%
HY-N0240	Herbacetin	4%	13%
HY-N0139	Troxerutin	3%	6%
HY-14590	Kaempferol	0%	25%
HY-14615	[6]-Gingerol	1%	10%
HY-136064	4,4'-Dimethoxychalcone	24%	3%
HY-22024	5-Hydroxyflavone	12%	21%
HY-N0002	(-)-Epicatechin gallate	5%	8%
HY-13065	Isobavachalcone	6%	15%
HY-N0154	Neohesperidin dihydrochalcone	10%	2%
HY-N0399	Wogonoside	6%	2%
HY-14589	Chrysin	15%	0%
HY-N0270	Ononin	10%	0%
HY-N2495	Isomucronulatol	7%	7%
HY-N4119	Neoeriocitrin	6%	13%
HY-13930	Miquelianin	1%	0%
HY-N0015	Astragalin	1%	1%
HY-111928	5,7-Dimethoxyluteolin	24%	13%
HY-N2278	Kushenol A	14%	10%
HY-N6819	Iristectorin B	5%	6%
HY-N2609	7,4'-Dihydroxyflavone	5%	0%
HY-N2258	Poncirin	13%	3%
HY-N0943	(-)-Epiafzelechin	13%	42%
HY-129529	6-Hydroxyluteolin 7-glucoside	0%	10%
HY-N2186	Leucoside	9%	8%
HY-N0779	Isosilybin	2%	0%

HY-N5010	Nepitrin	8%	0%
HY-N2524	Camelliaside A	9%	10%
HY-N0101	Neohesperidin	14%	0%
HY-N6009	8-Deoxygartanin	14%	0%
HY-N6820	Iristectorin A	0%	2%
HY-N1979	4'-Methoxypuerarin	14%	3%
HY-N0178	Diosmin	13%	4%
HY-N0616	Trifolirhizin	11%	0%
HY-N2122	Neoisoliquiritin	17%	11%
HY-N0168	Hesperetin	21%	5%
HY-N3847	Eriodictyol-7-O-glucoside	11%	4%
HY-N0102	Isoliquiritigenin	16%	4%
HY-N2153	Apigenin-7-O-(2G-rhamnosyl)gentiobioside	10%	0%
HY-N2438	Methylophiopogonanone B	0%	21%
HY-N0208	Kaempferol-3-O-glucorhamnoside	5%	2%
HY-N2008	Luteolin 5-O-glucoside	8%	0%
HY-N0794	Proanthocyanidins	7%	12%
HY-114336	Enocyanin	1%	12%
HY-N0355	(+)-Catechin hydrate	8%	8%
HY-N0938	Safflower yellow	3%	0%
HY-N0615	Notoginsenoside R1	5%	26%
HY-N0803	Myrcene	8%	23%
HY-N0038	Alantolactone	6%	29%
HY-N6258	Kahweol	11%	19%
HY-N0757	8-O-Acetylharpagide	10%	29%
HY-N0781	Linderalactone	0%	45%
HY-N4210	Yadanziolide A	0%	36%
HY-N2039	20(R)-Ginsenoside Rg2	0%	8%
HY-N2006	Ganoderic acid B	0%	20%
HY-N0475	Triptophenolide	11%	40%
HY-N0532	Morroniside	5%	42%
HY-N6075	Specneuzhenide	3%	31%
HY-N0331	Ziyuglycoside I	0%	6%
HY-N2057	Steviol	13%	34%
HY-Z0478	(-)-Limonene	12%	28%
HY-N2251	Hosenkoside C	0%	5%
HY-N2109	Macranthoidin A	1%	1%
HY-N0541	Pseudoginsenoside F11	0%	22%
HY-N0129	Sclareolide	1%	25%
HY-N0896	Inulicin	0%	16%
HY-N2205	Esculentoside H	0%	17%
HY-N0594	Deacetylasperulosidic Acid	0%	8%
HY-N0587	Demethylzeylasteral	0%	36%
HY-N0864	Macranthoidin B	6%	0%

HY-N4096	Tsugaric acid A	12%	12%
HY-N2518	Agnuside	2%	15%
HY-32736	Triptonide	7%	13%
HY-N2017	Maoecrystal A	7%	2%
HY-N1469	Kaurenoic acid	0%	7%
HY-N6792	T-2 Toxin	11%	2%
HY-N0906	Ginsenoside Rk3	0%	19%
HY-N0698	Crocin II	27%	1%
HY-N1382	Asperuloside	0%	5%
HY-N0204	Pulchinenoside A	15%	7%
HY-N0602	Ginsenoside Rg2	8%	0%
HY-N0865	Ingenol	1%	0%
HY-N6662	(+)-Longifolene	3%	1%
HY-N0665	Specnuezhenide	1%	13%
HY-N0852	Benzoylpaeoniflorin	10%	0%
HY-B1761	Santonin	37%	17%
HY-N0591	Dehydrocostus Lactone	0%	24%
HY-N0316	Mollugin	1%	12%
HY-N6862	Lucideric acid A	0%	20%
HY-N1484	7beta-Hydroxylathyrol	6%	14%
HY-N8314	8-Epiloganic acid	4%	15%
HY-N2112	Glaucocalyxin A	0%	7%
HY-N1423A	Glycocholic acid (sodium)	0%	0%
HY-13067	Celastrol	0%	13%
HY-N0332	Ziyuglycoside II	11%	14%
HY-N0396	Harpagoside	9%	30%
HY-N4289	3-Epiursolic Acid	3%	18%
HY-N0140	Ursolic acid	0%	0%
HY-N0380	Nardosinone	0%	13%
HY-N1490	14-Deoxy-11,12-didehydroandrographolide	0%	6%
HY-N6952	Geraniol	0%	3%
HY-N6073	Soyasapogenol A	5%	3%
HY-N0542	Pseudoginsenoside RT5	0%	16%
HY-16941	Damulin A	5%	0%
HY-124257	(R)-Citronellol	1%	14%
HY-N0084	Betulinaldehyde	0%	9%
HY-N0037	Albiflorin	0%	2%
HY-N1988	Cucurbitacin IIa	0%	6%
HY-108943	Sabinene	0%	20%
HY-N2031	Parishin	0%	12%
HY-N0718	Valepotriate	0%	7%
HY-N6947	Lutein	13%	0%
HY-N7665	Prosaikogenin G	2%	34%
HY-N0254	Hederacoside D	2%	2%

HY-N4214	Isoastragaloside IV	0%	0%
HY-N6850	Calenduloside E	0%	0%
HY-N0559	Kirenol	0%	15%
HY-N8144	Niga-ichigoside F1	0%	1%
HY-N0847	Micheliolide	0%	0%
HY-N3463	Isopimaric acid	0%	14%
HY-N0402	Artemether	1%	11%
HY-17411	Limonin	4%	15%
HY-N0870	Ingenol-5,20-acetonide-3-O-angelate	0%	0%
HY-N1984	Artemisic acid	1%	14%
HY-N2515	Ginsenoside Rk1	4%	0%
HY-N2070	Acevaltrate	7%	21%
HY-N0141	Parthenolide	6%	26%
HY-100560	Abscisic acid	0%	11%
HY-N1411	Platycodin D	0%	15%
HY-N0512	Loganin	11%	15%
HY-N1376	(20R)-Ginsenoside Rg3	22%	15%
HY-N1401	20(R)-Ginsenoside Rh2	11%	6%
HY-N0205	Pulchinenoside C	4%	0%
HY-B1173	(+)-Camphor	0%	0%
HY-17387	(-)-Huperzine A	0%	15%
HY-N0246	Saikosaponin A	2%	4%
HY-N0250	Saikosaponin D	34%	52%
HY-B0015	Paclitaxel	29%	23%
HY-N0180	18β-Glycyrrhetinic acid	6%	11%
HY-N0820	Catalpol	0%	16%
HY-N0908	Ginsenoside Rg5	0%	2%
HY-N0280	Corosolic acid	23%	34%
HY-N6998	Paederosidic acid	0%	0%
HY-N0784	Ginkgolide B	4%	7%
HY-10529	Betulinic acid	22%	31%
HY-N0424	Cyclogalegenin	22%	9%
HY-N0203	Atractylenolide III	3%	6%
HY-N0043	Ginsenoside Rd	4%	4%
HY-111664	(R)-(+)-Citronellal	0%	8%
HY-N0702	Tenuifolin	0%	1%
HY-N8211	Gypenoside L	22%	15%
HY-N0128	Sclareol	28%	10%
HY-N1072	Wilforgine	12%	5%
HY-N0407	Picroside I	6%	17%
HY-N2147	Phorbol	0%	6%
HY-N6036	Ganoderic acid F	14%	2%
HY-N0909	Notoginsenoside R2	19%	9%
HY-N0306	Hederasaponin B	11%	3%

HY-17389	Genipin	0%	0%
HY-119720	Neocryptotanshinone	18%	25%
HY-16942	Damulin B	9%	16%
HY-N2381	Menthone	17%	17%
HY-N0500	Mogroside III	7%	3%
HY-N9496	Piperitone	10%	11%
HY-N2565	Rosamultin	21%	1%
HY-N6928	Mogroside III-E	25%	17%
HY-N6778	Paxilline	0%	8%
HY-N0603	20(S)-Ginsenoside Rg3	0%	0%
HY-N0629	Maslinic acid	0%	5%
HY-N0805	Alisol B 23-acetate	0%	2%
HY-N6939	Pseudolaric Acid B	0%	9%
HY-N0042	Ginsenoside Rc	0%	0%
HY-N0808	Camphor	0%	10%
HY-N0004	Oridonin	0%	3%
HY-B0719	Ingenol Mebutate	1%	0%
HY-N0201	Atractylenolide I	0%	3%
HY-75161	(-)-Menthol	6%	0%
HY-N0156	Oleanolic Acid	1%	0%
HY-N0892	AKBA	0%	6%
HY-N0184	Glycyrrhizic acid	0%	0%
HY-N1214	Squalene	4%	19%
HY-B0094	Artemisinin	0%	0%
HY-N0242	Fraxinellone	0%	6%
HY-N2447	Amarogentin	2%	8%
HY-N6985	Baccatin III	0%	0%
HY-N0045	Ginsenoside Rg1	0%	0%
HY-N0036	Costunolide	0%	21%
HY-N0255	alpha-Hederin	6%	0%
HY-N2178	Saikosaponin F	1%	0%
HY-N2243	Hosenkoside K	10%	2%
HY-N0408	Picroside II	0%	13%
HY-N0330	Momordin Ic	12%	4%
HY-N0009	Geniposide	0%	0%
HY-N0871	Ingenol-3,4,5,20-diacetonide	1%	9%
HY-122550	Artemisitene	0%	9%
HY-N0566	23-Hydroxybetulinic acid	13%	5%
HY-126741	Azadirachtin	24%	4%
HY-N0202	Atractylenolide II	10%	2%
HY-N2499	Dehydrotumulosic acid	11%	21%
HY-N4176	Ginkgolide K	0%	2%
HY-N1391	10-Deacetyltaxol	17%	0%
HY-N2015	Hastatoside	0%	8%

HY-N0249	Saikosaponin C	24%	0%
HY-N1478	Gardenoside	0%	8%
HY-N0818	Chikusetsusaponin Iva	5%	14%
HY-N2993	Polyporenic acid C	30%	12%
HY-N2163	Mudanpioside C	23%	41%
HY-N0842	Bevirimat	3%	30%
HY-N2085	L-Perillaldehyde	22%	25%
HY-N2097	Cimigenoside	30%	17%
HY-N4132	17-Hydroxyisolathyrol	13%	29%
HY-N2151	Rhodojaponin II	4%	8%
HY-N2170	Acetylshengmanol Arabinoside	30%	30%
HY-N3026	Soyasaponin Ab	21%	4%
HY-N3005	Britannin	14%	5%
HY-N4237	Saikogenin D	27%	14%
HY-42937	Crocetin (meglumine)	6%	10%
HY-N3037	3-O-Acetyl-α-boswellic acid	20%	14%
HY-N6992	Dulcoside A	0%	11%
HY-N0468	Rebaudioside D	11%	3%
HY-N2169	Polygalasaponin V	9%	0%
HY-125938	Cycloartenyl ferulate	35%	21%
HY-N1474	Picfeltarraenin IA	5%	2%
HY-N1486	Ursonic acid	18%	23%
HY-N0562	5,15-Diacetyl-3-benzoyllathyrol	30%	32%
HY-N2184	Furanodienone	0%	0%
HY-N6808	Rebaudioside B	6%	1%
HY-N0732	Jolkinolide B	18%	15%
HY-N1502	Carboxyatractyloside (tripotassium)	23%	23%
HY-N0310	Soyasaponin Bb	0%	0%
HY-N2453	Convallatoxin	2%	3%
HY-N8102	Echinocystic acid 28-O-β-D-glucoside	36%	41%
HY-N0598	Ginsenoside F1	10%	2%
HY-N0588	Deapioplatycodin D	12%	0%
HY-N0888	Isoastragaloside II	0%	9%
HY-N6908	Continentalic acid	36%	24%
HY-N0553	Gypenoside XVII	4%	4%
HY-N1451	Betulonic acid	24%	26%
HY-N0853A	Alisol A 24-acetate	3%	23%
HY-N4218	Saikosaponin B4	13%	36%
HY-N7136	α-Terpinyl acetate	18%	28%
HY-N0271	Echinocystic acid	45%	35%
HY-N0907	Ginsenoside Rg6	1%	4%
HY-N0673	Pseudolaric Acid A	6%	13%
HY-N1990	Gypenoside XLIX	3%	6%
HY-N2249	Hosenkoside A	15%	19%

HY-N0547	Nomilin	0%	22%
HY-N4246	Bacopaside I	19%	0%
HY-N2815	Ursolic acid acetate	24%	18%
HY-N1964	Gibberellic acid	17%	22%
HY-N5132	(-)-Fenchone	26%	22%
HY-N0513	Loganic acid	28%	29%
HY-N0688	Linderane	5%	16%
HY-N5142	α-Terpineol	22%	17%
HY-129434A	4aα,7α,7aα-Nepetalactone	17%	16%
HY-N0835	(20S)-Protopanaxatriol	31%	32%
HY-N6902	Linderene acetate	8%	20%
HY-N8118	Euphorbia factor L7b	14%	37%
HY-N2666	5α-Hydroxycostic acid	13%	18%
HY-N0416	Cucurbitacin B	5%	0%
HY-N0557	Isoescin IB	9%	0%
HY-N2590	Lupenone	25%	17%
HY-N6843	Arnicolide D	16%	25%
HY-N2541	Gymnemic acid I	20%	0%
HY-N0672	Pseudolaric Acid C	31%	42%
HY-N2099	Onjisaponin B	30%	33%
HY-N2312	Mogrol	5%	2%
HY-N7616	Oleanolic acid-3-O-glucosyl(1-2)xylyl(1-3)glucosiduronic acid	0%	0%
HY-N2100	6'-O-beta-D-Glucosylgentiopicroside	34%	15%
HY-N2547	Steviolbioside	10%	5%
HY-76225	Ammonium glycyrrhizinate	20%	11%
HY-N7117	1,4-Cineole	21%	35%
HY-N2456	Mogroside IV-E	4%	6%
HY-N10321	Kanshone C	17%	22%
HY-N0676	Dehydroandrographolide	4%	9%
HY-125361	Ganosporeric acid A	36%	9%
HY-N4290	3-Epioleanolic acid	29%	34%
HY-N1487	Oleanonic acid	15%	26%
HY-N7667	Prosaikogenin D	15%	54%
HY-N7924	Astragenol	50%	50%
HY-N1404	Sodium aescinate	33%	31%
HY-N6815	Isomogroside V	28%	21%
HY-N3557	Cauloside A	12%	24%
HY-N0458	Pedunculoside	14%	13%
HY-N0845	Epoxymicheliolide	16%	24%
HY-N6967	Levomenol	37%	50%
HY-N0819	Raddeanin A	4%	6%
HY-N5140	Bacopaside X	9%	35%
HY-N2250	Hosenkoside B	7%	10%

HY-N2522	Carboxyatractyloside (dipotassium)	15%	18%
HY-N2195	Nootkatone	28%	20%
HY-N8534	Neophytadiene	3%	6%
HY-N2115	Araloside A	6%	0%
HY-N6265	Cauloside F	8%	0%
HY-N7026	Celosin I	4%	0%
HY-N0041	Ginsenoside Rb3	6%	1%
HY-N0440	Germacrone	12%	9%
HY-N0223	Epibetulinic acid	14%	17%
HY-N0659	Jujuboside A	0%	0%
HY-N0263	Toosendanin	0%	7%
HY-N0785	Ginkgolide C	12%	0%
HY-115363	Curcumenone	3%	0%
HY-N0549	(-)-α-Pinene	0%	6%
HY-N7691	3,29-O-Dibenzoyloxykarounidiol	15%	9%
HY-N8290	Lactupicrin	7%	25%
HY-N0439	Asiaticoside	6%	0%
HY-32735	Triptolide	15%	72%
HY-N1919	Ajmalicine	0%	1%
HY-N1932	Bayogenin	2%	5%
HY-N3697	Demethoxydeacetoxypseudolaric acid B	0%	0%
HY-N6661	Verbenone	4%	4%
HY-N3651	Curzerenone	10%	9%
HY-N4137	Tormentic acid	3%	5%
HY-N0375	18α-Glycyrrhetinic acid	2%	10%
HY-N0044	Ginsenoside Re	0%	0%
HY-N4092	Shanziside	1%	4%
HY-N1991	Platicodigenin	0%	0%
HY-N0409	Picroside III	3%	7%
HY-N0607	Ginsenoside Ro	1%	0%
HY-N0677	Dehydroandrographolide succinate	0%	0%
HY-N0597	Panaxatriol	5%	11%
HY-B0355	Ginkgolide A	2%	8%
HY-N1467	(-)-α-Terpineol	15%	17%
HY-N6804	Diammonium Glycyrrhizinate	0%	11%
HY-N0176	Dihydroartemisinin	9%	13%
HY-N2991	Dehydropachymic acid	29%	35%
HY-N6920	11-Oxomogroside IIIE	6%	1%
HY-N0426	Alismoxide	2%	0%
HY-N0502	Mogroside V	19%	12%
HY-N2241	Hosenkoside F	12%	0%
HY-N2016	Arteannuin B	13%	11%
HY-N0612	Siamenoside I	16%	10%
HY-N0561	Lathyrol	1%	3%

HY-107324	β-Elemene	7%	2%
HY-N1500	Pulegone	6%	5%
HY-N7652	Terminolic acid	26%	10%
HY-N0669	Stevioside	15%	0%
HY-N0664	Aucubin	10%	19%
HY-N0869	Ingenol-5,20-acetonide	4%	6%
HY-N0206	Ardisiacrispin A	22%	0%
HY-N8198	Ardisiacrispin B	16%	0%
HY-110028	Leelamine (hydrochloride)	22%	57%
HY-N2244	Hosenkoside M	5%	0%
HY-N6636	Valencene	3%	4%
HY-N0253	Hederacoside C	18%	0%
HY-B2114	Escin	23%	6%
HY-N4098	Incensole Acetate	9%	17%
HY-N5064	Bacoside A3	3%	0%
HY-135013	Umbellulone	0%	1%
HY-N4124	Poricoic acid B	14%	0%
HY-N2152	Rhodojaponin III	5%	2%
HY-N2138	Buddlejasaponin IVb	0%	4%
HY-N9522	β-D-glucopyranosyl-[α-L-rhamnopyranosyl-(1→3)-βD-glucuronopyranosyl-(1→3)]-3β-hydroxyolean-12-ene28-oate	6%	0%
HY-N3075	Phytol	13%	5%
HY-N6833	Rebaudioside M	7%	0%
HY-N0397	Harpagide	9%	13%
HY-N0266	Dipsacoside B	7%	5%
HY-N1447	Ganoderic acid A	15%	6%
HY-16565	10-Deacetylbaccatin III	4%	5%
HY-N3001	Isolinderalactone	13%	9%
HY-N0568	Madecassoside	12%	1%
HY-N6951	Guaiazulene	4%	12%
HY-N0839	Quillaic acid	14%	16%
HY-N0193	Artesunate	21%	15%
HY-N3544	Caryophyllene oxide	13%	13%
HY-N0432	Astragaloside I	13%	23%
HY-N4165	Arnidiol	12%	57%
HY-B1350A	Fusidic acid (sodium salt)	0%	44%
HY-N2268	Gymnemagenin	0%	52%
HY-N6855	Mogroside IIA1	6%	47%
HY-N0313	Euphol	0%	41%
HY-N1987	Cucurbitacin IIb	7%	43%
HY-20584	10-Deacetyl-7-xylosyl paclitaxel	0%	14%
HY-15371	Forskolin	0%	2%
HY-122951	Eburicoic acid	27%	56%

HY-N2419	Erythrodiol	11%	68%
HY-101795	Larixyl acetate	0%	44%
HY-111664A	(S)-(-)-Citronellal	18%	61%
HY-N0900	Cimiracemoside D	0%	0%
HY-N1921	Edpetiline	11%	56%
HY-N6015	Bacopasaponin C	6%	40%
HY-16502	Crocetin (disodium)	0%	5%
HY-N0679	Retinyl acetate	0%	0%
HY-N0237	Atractyloside A	13%	28%
HY-N0630	Shanzhiside methyl ester	32%	38%
HY-N2472	Medicagenic acid	0%	5%
HY-116514	(S)-(−)-Perillyl alcohol	36%	35%
HY-N4323	14-Deoxyandrographolide	1%	28%
HY-N8187	Eupalinolide O	1%	9%
HY-N5037	Ilexoside D	0%	22%
HY-N2282	Zingiberen Newsaponin	0%	60%
HY-77572	7-Epi 10-desacetyl paclitaxel	0%	26%
HY-N4215	11(α)-Methoxysaikosaponin F	0%	7%
HY-136150	Methyl carnosate	0%	63%
HY-N0207	Patchouli alcohol	24%	35%
HY-N2014	Verbenalin	60%	44%
HY-N1462	Atractyloside (potassium salt)	29%	45%
HY-N2223	Ganoderol B	5%	35%
HY-N3181	Nodosin	0%	14%
HY-N0829	Shionone	24%	52%
HY-N6911	Licorice-saponin H2	0%	14%
HY-N0501	11-oxo-mogroside V	3%	24%
HY-N0429	Diosbulbin B	0%	31%
HY-N10096	Epoxyazadiradione	19%	47%
HY-N6945	Mogroside IV	0%	43%
HY-N7495	all-trans-Anhydro Retinol	61%	58%
HY-N1384	Ambroxide	8%	14%
HY-107738	Guggulsterone	0%	17%
HY-N0433	Astragaloside II	5%	45%
HY-N0841	Bruceine A	24%	49%
HY-113342	7-Ketocholesterol	1%	27%
HY-N2638	Ilexsaponin A	0%	12%
HY-N4121	Isocurcumenol	5%	24%
HY-N7422	Kauran-16,17-diol	0%	22%
HY-N6851	Glycyrrhetic acid 3-O-β-D-glucuronide	2%	22%
HY-N6252	Gypenoside XLVI	1%	2%
HY-N6874	Ligustrosidic acid	3%	24%
HY-125848	Ginsenoside F2	0%	45%
HY-N0613	Sauchinone	18%	25%

HY-N0010	Geniposidic acid	0%	3%
HY-N7510	12-O-Methylcarnosic acid	5%	11%
HY-N6878	Cauloside D	6%	17%
HY-N0555	Escin IB	0%	0%
HY-N0434	Astragaloside III	0%	35%
HY-107242	Raddeanoside R8	3%	0%
HY-17388	(±)-Huperzine A	0%	16%
HY-N4325	Onjisaponin Z	4%	21%
HY-N2061	Lindenenol	0%	19%
HY-N0481	Roburic acid	11%	15%
HY-N4097	Incensole	2%	29%
HY-N1503	Methyl deacetylasperulosidate	16%	30%
HY-N2556	Tirucallol	12%	39%
HY-41094	cis-Isolimonenol	2%	30%
HY-N1388	Tussilagone	12%	31%
HY-N1485	Cycloastragenol	8%	31%
HY-N6968	α-Humulene	11%	55%
HY-W024698	β-Cyclogeraniol	15%	48%
HY-N1986	Cucurbitacin D	10%	22%
HY-N0721	Neoandrographolide	15%	40%
HY-N2566	Euscaphic acid	18%	27%
HY-N2530	Notoginsenoside Fa	7%	33%
HY-N2454	β-Elemonic acid	23%	28%
HY-N8537	Enfumafungin	0%	60%
HY-N0904	Ginsenoside C-K	4%	22%
HY-N6601	Pomolic acid	29%	67%
HY-N0357	3,29-Dibenzoyl rarounitriol	40%	71%
HY-N9377	Platyconic acid A	0%	5%
HY-N9506	Anisatin	0%	20%
HY-N6074	Soyasapogenol B	9%	39%
HY-N2211	Picfeltarraenin IB	0%	34%
HY-N0780	Isoalantolactone	2%	12%
HY-N0494	Gentiopicroside	0%	19%
HY-N0076	Bilobalide	0%	23%
HY-135190	(-)-Cedrene	0%	17%
HY-N0786	Ginkgolide J	0%	25%
HY-N5081	1αH,5αH-Guaia-6-ene-4β,10β-diol	0%	27%
HY-N2995	Poricoic acid A	2%	8%
HY-N4087	Platycodin D2	0%	2%
HY-N3523	3-O-Beta-D-Glucopyranosylplatycodigenin	0%	0%
HY-N0891	Tubeimoside II	0%	2%
HY-N6873	GL3	0%	18%
HY-N0915	Melittoside	0%	4%
HY-N2998	Ganoderenic acid A	0%	0%

HY-N0569	Madecassic acid	1%	31%
HY-N6915	Mogroside II-A	0%	14%
HY-N4171	Dihydrocucurbitacin B	0%	11%
HY-N0554	Escin IA	0%	0%
HY-N0604	Ginsenoside Rh1	0%	9%
HY-N0467	Rebaudioside C	0%	6%
HY-N0798	20(R)-Protopanaxatriol	0%	21%
HY-N2423	Sinigrin (hydrate)	0%	19%
HY-N7608	Beiwutine	0%	10%
HY-N9331	Cimicifugoside H-1	0%	3%
HY-N6018	Beta-Eudesmol	0%	6%
HY-N2261	Ipecoside	0%	14%
HY-N1479	Polygalic acid	0%	1%
HY-N0412	Sesamoside	4%	25%
HY-N0631	Cornuside	0%	24%
HY-N6257	Cafestol	0%	28%
HY-N0309	Soyasaponin Ba	0%	25%
HY-N2994	3-O-Acetyl-16α-hydroxytrametenolic acid	7%	10%
HY-N6875	Oleuropeinic acid	0%	8%
HY-N0389	Columbin	0%	1%
HY-117469	Triptohypol C	0%	43%
HY-N9337	Amaroswerin	0%	0%
HY-N2400	Rehmannioside C	0%	8%
HY-N9357	Granilin	4%	13%
HY-N0167	Gynostemma Extract	0%	4%
HY-N0887	Isoastragaloside I	12%	59%
HY-N0912	Rehmannioside D	11%	35%
HY-N3014	Bruceine D	25%	54%
HY-N2177	3-Dehydrotrametenolic acid	17%	37%
HY-120140	Ganoderic acid DM	6%	1%
HY-123100	Ganoderic acid N	0%	0%
HY-113074	Glycolithocholic acid 3-sulfate	13%	33%
HY-N2542	Tubeimoside III	0%	36%
HY-N2772	8-Epideoxyloganic acid	2%	17%
HY-N5012	Eurycomanone	0%	4%
HY-N2417	Stearyl glycyrrhetinate	0%	0%
HY-N0754	Eupalinolide A	11%	38%
HY-N1943	Ailanthone	31%	65%
HY-N0854	Alisol F	0%	11%
HY-N6942	Mogroside IV-A	0%	0%
HY-N0853	Alisol A	3%	2%
HY-N7640	Deoxylimonin	9%	50%
HY-N6890	Tarasaponin VI	0%	0%
HY-N7899	11-Deoxymogroside V	0%	10%

HY-N7592	Arjunetin	0%	18%
HY-N8122	24-Methylenecycloartanyl ferulate	0%	11%
HY-136552	β-Bisabolene	8%	57%
HY-N7363	Isolongifolene	2%	39%
HY-N6814	Mogroside IIe	0%	7%
HY-N2491	Deoxyelephantopin	0%	9%
HY-N2999	Ganoderic acid I	0%	11%
HY-N4294	Arjungenin	0%	36%
HY-N0840	Bruceantin	24%	69%
HY-W020183	γ-Terpinene	6%	31%
HY-N0660	Jujuboside B	0%	0%
HY-N0668	Rubusoside	0%	18%
HY-N2259	Curcumenol	0%	38%
HY-N6028	Darutoside	2%	9%
HY-N4231	Lucyoside B	9%	4%
HY-N2458	Ganoderic acid G	0%	3%
HY-N0600	Ginsenoside F3	2%	34%
HY-N2040	(20R)-Protopanaxadiol	0%	43%
HY-77554	Cephalomannine	31%	34%
HY-N1951	Miltirone	0%	36%
HY-N6938	Pseudolaric acid B β-D-glucoside	4%	0%
HY-N2531	Notoginsenoside Fc	0%	0%
HY-N6776	Penitrem A	0%	14%
HY-N1405	Cucurbitacin I	0%	23%
HY-N7615	Momordin IIc	0%	0%
HY-N8207	Gypenoside LI	0%	0%
HY-N0601	Ginsenoside Rf	0%	0%
HY-N0417	Cucurbitacin E	0%	11%
HY-N7497	Protoescigenin	27%	41%
HY-N3009	Secoxyloganin	0%	3%
HY-N4106	Dihydroartemisinic acid	0%	15%
HY-N5052	Asiaticoside B	0%	5%
HY-N9388	Andrograpanin	3%	20%
HY-N7364	(E)-β-Farnesene	0%	11%
HY-N2506	Ginsenoside Ra1	0%	0%
HY-N3373	Loganetin	0%	2%
HY-N1511	Ganoderic acid D	0%	21%
HY-N1433	Phytolaccagenin	5%	15%
HY-N4135	Acanthopanaxoside B	0%	0%
HY-N6016	Bacopaside II	7%	9%
HY-N0872	Isosteviol	0%	2%
HY-N7000	Perillyl alcohol	0%	6%
HY-N0227	7-epi-Taxol	13%	11%
HY-N6881	Gypenoside XIII	7%	13%

HY-N0265	Asperosaponin VI	0%	2%
HY-N0753	Eupalinolide B	0%	0%
HY-N7629	Lactiflorin	0%	11%
HY-N1514	Ganoderenic acid B	0%	42%
HY-N0307	Ciwujianoside B	0%	0%
HY-N2221	Ganoderal A	7%	25%
HY-N0104	Curcumol	8%	12%
HY-N0856	Alisol C 23-acetate	0%	17%
HY-N7819	Pristane	8%	20%
HY-N6046	Kamebakaurin	0%	11%
HY-N2440	Gypenoside A	0%	0%
HY-N0834	Pulsatilla saponin D	0%	0%
HY-N6983	Licoricesaponin G2	2%	10%
HY-N6869	Dehydroabietic acid	4%	17%
HY-N2075	3-Acetyl-beta-boswellic acid	15%	21%
HY-N6051	(-)-Maackiain	26%	26%
HY-N0866	20-Deoxyingenol	0%	21%
HY-N6919	Cauloside C	0%	10%
HY-N1937	Pristimerin	15%	24%
HY-N5001	Euphorbia Factor L2	27%	21%
HY-N1371	Clinodiside A	1%	0%
HY-N2217	Rotundic acid	24%	45%
HY-108450	Polygodial	0%	93%
HY-N3016	Rupestonic acid	0%	8%
HY-111896	7-Methoxyrosmanol	12%	18%
HY-N7635	Oleanolic acid 28-O-β-D-glucopyranoside	33%	34%
HY-N4277	Methyl protogracillin	0%	15%
HY-121222	alpha-Bisabolol	1%	6%
HY-N0697	Crocin	11%	11%
HY-N7162	3-O-Acetyl-11-hydroxy-beta-boswellic acid	24%	23%
HY-N2083	Handelin	0%	14%
HY-N0805A	Alisol B	3%	10%
HY-125120	Kansuiphorin C	5%	29%
HY-N0556	Isoescin IA	8%	1%
HY-N8573	β-Phellandrene	17%	20%
HY-77434	9-Dihydro-13-acetylbaccatin III	0%	8%
HY-N2094	Genipin 1-β-D-gentiobioside	0%	17%
HY-121382	Gypsogenin	10%	10%
HY-N0428	Obacunone	2%	2%
HY-N2201	Pseudoginsenoside RT1	1%	0%
HY-N0353	Curdione	7%	22%
HY-N0801	Polygalacic acid	3%	0%
HY-N7041	11-Oxomogroside IIa	0%	0%
HY-N0463	Blinin	0%	7%

HY-N6910	Pseudolaric Acid C2	0%	0%
HY-N0611	alpha-Boswellic acid	34%	28%
HY-N6946	Mitraphylline	0%	21%
HY-139058	23-epi-26-Deoxyactein	0%	5%
HY-B1350	Fusidic acid	0%	1%
HY-N4125	Dehydroeburicoic acid monoacetate	20%	24%
HY-N0890	Tubeimoside I	3%	0%
HY-N0466	Rebaudioside A	0%	12%
HY-N4088	Pseudolaric acid A-O-β-D-glucopyranoside	0%	2%
HY-125703	Ferutinin	0%	16%
HY-N0605	Ginsenoside Rh2	34%	38%
HY-108277	Ginsenoside F5	43%	17%
HY-121401A	(−)-Myrtenal	21%	14%
HY-N6255	Ilexgenin A	27%	0%
HY-N0632	Esculentoside A	17%	3%
HY-N4312	Bryodulcosigenin	16%	15%
HY-N0256	Hederagenin	30%	21%
HY-N6076	Tenuifoliside A	0%	15%
HY-N0293	Paeoniflorin	4%	10%
HY-N0802	Tenuigenin	11%	11%
HY-N0596	Panaxadiol	39%	36%
HY-N7670	Saikogenin F	53%	46%
HY-121618	α-Thujone	9%	20%
HY-107782	Picrotin	0%	0%
HY-N1439	Gypsogenin-3-O-glucuronide	50%	10%
HY-N0758	Barlerin	12%	26%
HY-N1400	(20R)-Ginsenoside Rh1	13%	5%
HY-N2056	11-Keto-beta-boswellic acid	2%	0%
HY-N0914	Ajugol	0%	2%
HY-N0899	Wilforine	23%	9%
HY-N0191	Andrographolide	2%	19%
HY-N6246	Asperulosidic Acid	2%	0%
HY-N6950	Hederacolchiside A1	0%	0%
HY-N0292	Oleuropein	0%	0%
HY-N6577	Astragaloside VI	3%	0%
HY-N0748	Oxypaeoniflorin	0%	0%
HY-N4306	Sarracenin	11%	27%
HY-19543	Brusatol	48%	37%
HY-137295	Ingenol 3,20-dibenzoate	0%	0%
HY-N1535	Ponicidin	4%	0%
HY-N0797	(20S)-Protopanaxadiol	21%	10%
HY-N0194	Asiatic acid	41%	55%
HY-N0371	Pachymic acid	45%	21%
HY-N2959	Brevilin A	0%	0%

HY-N1508	Ecliptasaponin A	12%	31%
HY-W010201	Citronellol	15%	28%
HY-N0368	Linalool	14%	20%
HY-N8121	Euphorbia factor L7a	21%	23%
HY-N2513	β-Boswellic acid	32%	22%
HY-N2071	Cedrol	8%	13%
HY-N1415	β-Caryophyllene	28%	27%
HY-N0392	Polygalasaponin F	5%	0%
HY-N7022	Eclalbasaponin I	35%	35%
HY-N0040	Ginsenoside Rb2	7%	0%
HY-N0677A	Kalii Dehydrographolidi Succinas	5%	5%
HY-N0134	Tanshinone I	0%	3%
HY-N0248	Saikosaponin B2	19%	11%
HY-N2432	Paederoside	9%	4%
HY-N0046	Notoginsenoside Fe	0%	0%
HY-N0083	Betulin	0%	0%
HY-N0790	Lupeol	0%	0%
HY-N0431	Astragaloside IV	10%	0%
HY-N0648	Monotropein	0%	0%
HY-N7624	Methyl oleanonate	4%	1%
HY-100597	Saponins	0%	0%
HY-N1944	Nerolidol	7%	7%
HY-N0184A	Dipotassium glycyrrhizinate	15%	3%
HY-N9437	Betulin diacetate	11%	0%
HY-N0039	Ginsenoside Rb1	4%	0%
HY-N7107	Fenchyl alcohol	2%	0%
HY-N0476	Wilforlide A	7%	3%
HY-N4157	Isopteropodine	9%	0%
HY-W040264	24,25-Dihydrolanosterol	3%	4%
HY-N6677	β-Apo-8'-carotenal	12%	1%
HY-N4233	Bisabolangelone	18%	13%
HY-126114	Lupeol acetate	0%	0%
HY-N0910	Notoginsenoside Ft1	14%	3%
HY-N1178	Taraxasterol	1%	2%
HY-N1109	Uvaol	19%	2%
HY-116035	Nimbolide	0%	0%

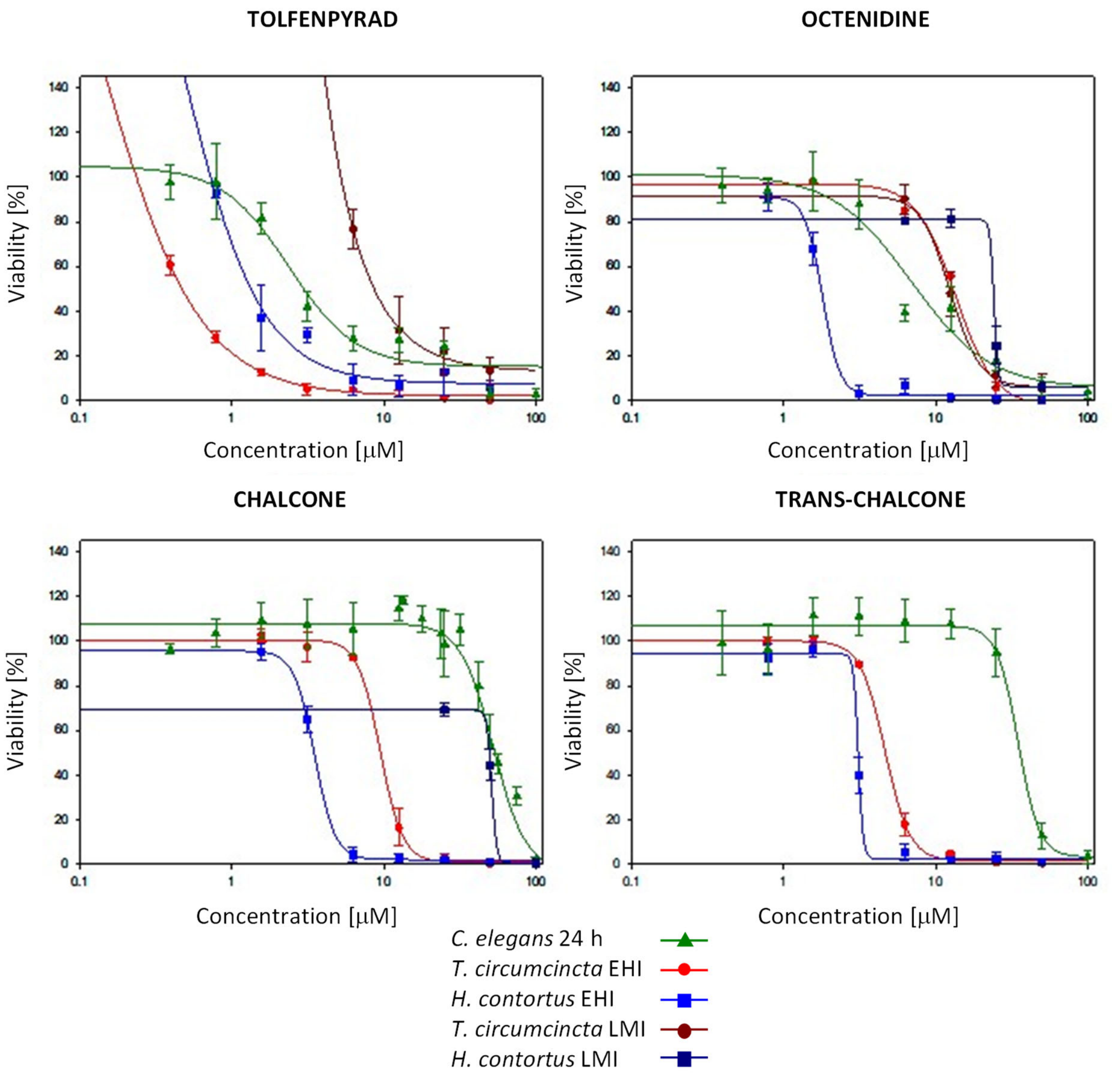

Figure S3. Dose-effect curves of the DR assay on *C. elegans* at 24 hours and of the EHI and LMI tests on *T. circumcicta* and *H. contortus*. Plots were elaborated with SigmaPlot® 10.0. Tolfenpyrad, octenidin, chalcone and trans-chalcone were tested at different concentrations ranging from 100 μM to 0.39 μM. Among the 32 compounds that reduced motility by more than 70% at 110 μM after 0 h and 24 h of exposure in the SS experiments (Table 1), only the dose-effects curves for tolfenpyrad, octenidin, chalcone and trans-chalcone are shown for *C. elegans*.

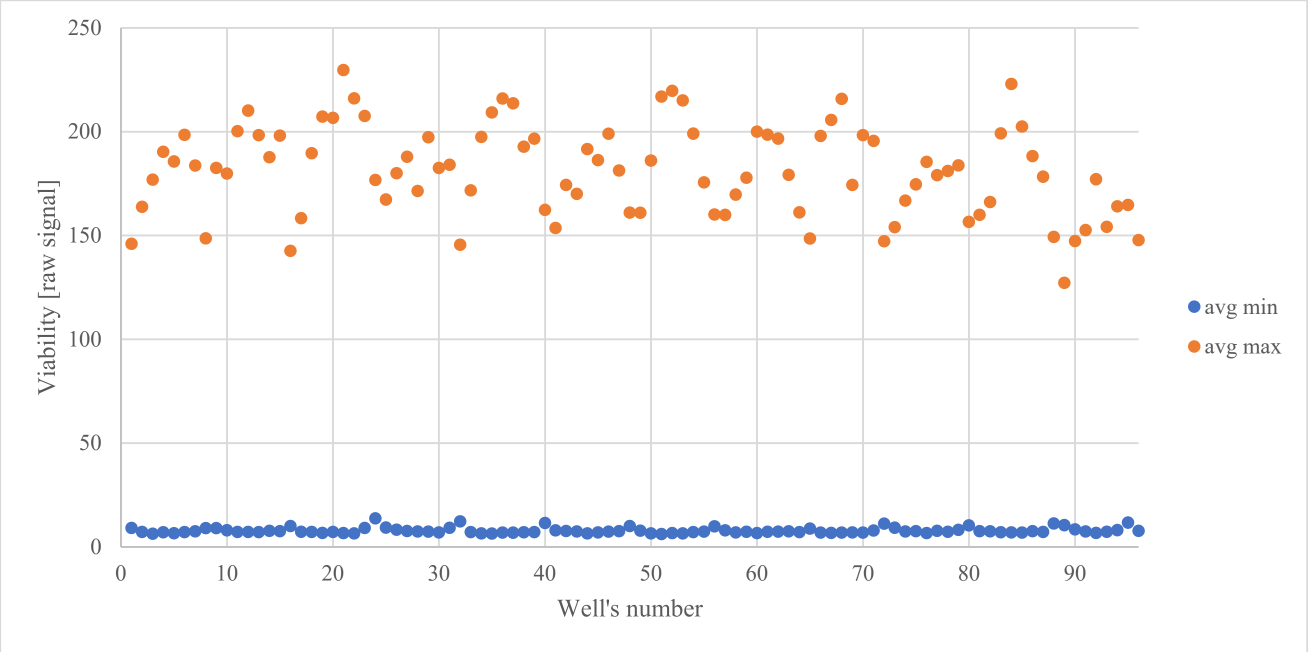

Figure S4. Raw signal per well recorded during Z′-factor test (averages) for the HepG2 spheroids assay.

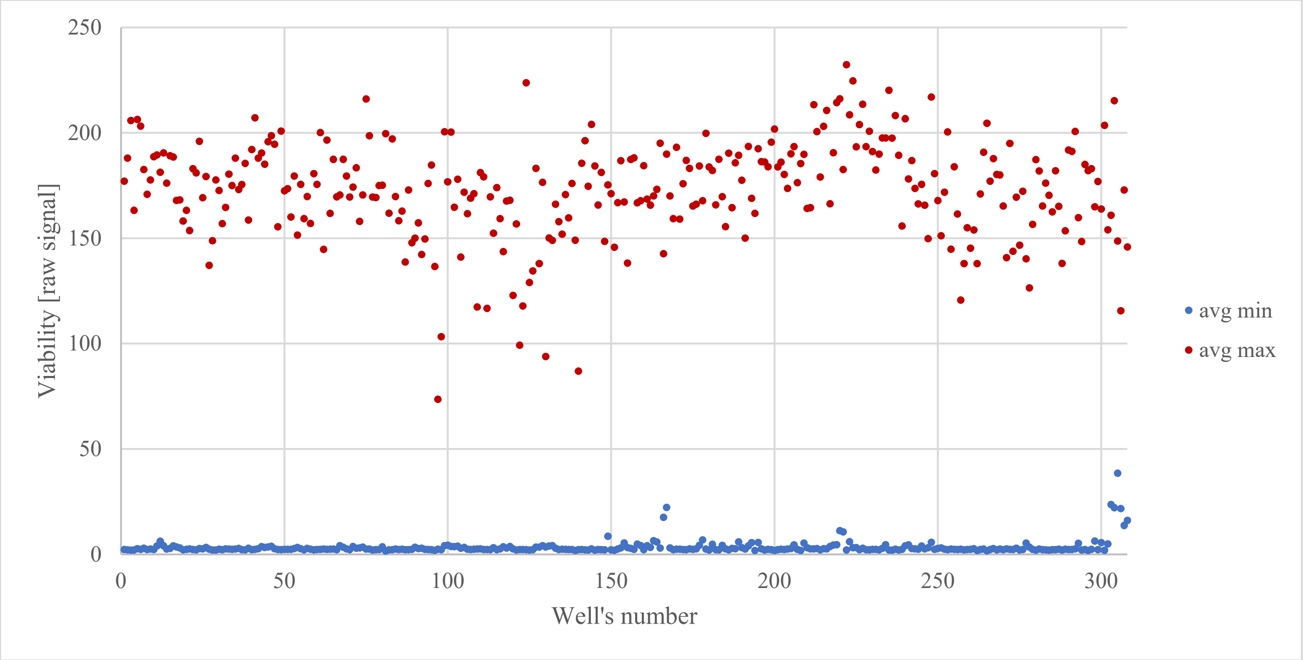

Figure S5. Raw signal per well recorded during Z′-factor test (averages) for the mouse enteroids assay.

Table S3. Values obtained from the Z′-factor test and respective averages for the HepG2 spheroids assay. P1-6 indicate 6 independent experiments in 3 different days. Reference values: Z′>0.5, SW>2, AVR<0.5.

	P1	P2	P3	P4	P5	P6	**Average**
**Z'**	0.6	0.5	0.6	0.6	0.5	0.6	0.6
**S/B**	18	31	24	18	32	26	25
**S/N**	67	300	133	75	232	189	166
**SW**	5.9	3.4	4.4	4.9	3.2	3.9	4.3
**AVR**	0.4	0.5	0.4	0.4	0.5	0.4	0.4

Table S4. Values obtained from the Z′-factor test and respective averages for the mouse enteroids assay. P1-6 indicate 6 independent experiments in 3 different days. Reference values: Z′>0.5, SW>2, AVR<0.5.

	P1	P2	P3	P4	**Average**
**S/B**	61	53	57	66	57
**S/N**	95	81	126	389	101
**Z'**	0.6	0.5	0.6	0.5	0.5
**SW**	4.5	2.7	4.0	2.7	3.7
**AVR**	0.4	0.5	0.4	0.5	0.5
